# The histone methyltransferase SETD2 regulates adult brain structure, connectivity and neurogenesis

**DOI:** 10.1038/s41598-025-18780-7

**Published:** 2025-09-29

**Authors:** Cooper Atterton, Hallie Naumann, Benjamin Mitchell, Bee Meibusch, Laura Currey, Nyoman D. Kurniawan, Stefan Thor, Michael Piper

**Affiliations:** 1 The School of Biomedical Sciences, Faculty of Health, Medicine and Behavioural Sciences, The University of Queensland, Brisbane, QLD 4072 Australia; 2 Australian Institute for Bioengineering and Nanotechnology, Centre for Advanced Imaging, The University of Queensland, Brisbane, QLD 4072 Australia; 3 The Queensland Brain Institute, Faculty of Health, Medicine and Behavioural Sciences, The University of Queensland, Brisbane, QLD 4072 Australia

**Keywords:** SETD2, DT-MRI, Hippocampus, Adult neurogenesis, Luscan-Lumish syndrome, Developmental biology, Neuroscience

## Abstract

Epigenetic mechanisms are crucial for neocortical development. Recent work has highlighted that ablation of *Setd2*, which encodes a SET domain lysine methyltransferase, from the mouse dorsal telencephalon results in deficits in reciprocal cortico-thalamic connectivity. However, analysis of broader cortical phenotypes in this model has yet to be performed. Here, we addressed this, revealing that dorsal telencephalon-specific ablation of *Setd2* results in decreased structural volumes across the entire adult brain, particularly within the hippocampus. Moreover, while homozygous *Setd2*-deficient brains exhibited apparently normal axonal integrity within the corpus callosum, axonal connections arising from the hippocampus were significantly impacted. Furthermore, whole brain structural connectivity was significantly altered in the absence of *Setd2*. Finally, we revealed structural, morphological and cellular changes within the hippocampal dentate gyrus of mutant mice. Collectively, these findings demonstrate a key role for SETD2 function in the development of the adult brain.

**Supplementary Information:**

The online version contains supplementary material available at 10.1038/s41598-025-18780-7.

## Introduction

Su(var)3–9, Enhancer of Zeste and Trithorax domain-containing protein 2 (SETD2) belongs to the nuclear receptor SET domain family of protein lysine methyltransferases^1,2^. Structurally, SETD2 contains several domains that are important for its catalytic activity, including the pre-SET or Associated with SET domain, SET and post-SET domains. SETD2 also contains several C-terminal domains important for protein–protein interactions, including the SETD2 hnRNP Interaction domain, Proline-rich region and Tryptophan-Tryptophan domains and the Set2 Rpb1 Interacting domain, which are important for its methyltransferase activity. SETD2 targets a number of substrates within the cell, but is most prominently associated with the deposition of the trimethylation mark on histone H3 at lysine residue 36 (H3K36me3)^2^. Trimethylation at H3K36 is crucial for the regulation of DNA methylation and H3K27me3 deposition, repair of DNA damage and repression of cryptic transcription^2^. Additional roles for SETD2 include interactions with RNA Polymerase II, methylation of actin and α-tubulin, and methylation of lysine residues on a number of different proteins including STAT1 and EZH2^1,2^.

SETD2 is expressed both during development, and in the adult^3–5^. In humans, the importance for SETD2 activity is underscored by the fact that loss of a single allele gives rise to the rare neurodevelopmental disorder, Luscan-Lumish syndrome (LLS)^6–8^. LLS falls under the category of disorders known as overgrowth-intellectual disability disorders (OGID), which typically include macrocephaly, bodily overgrowth, intellectual disability of a varying degree, and a range of other co-morbidities, including autism spectrum disorder (ASD), language and speech acquisition delays and brain malformations^6,9,10^. Many of the symptoms of LLS relate to nervous system structure, such as the presence of Chiari I malformations and function, including intellectual disability and ASD^6^. This suggests that SETD2 is important during embryonic and postnatal nervous system development, an idea further supported by the widespread expression of this methyltransferase within the developing brain^4,5^.

Despite this, our understanding of how SETD2 shapes neural development is limited. While SETD2 has been shown to be critical for development of systems such as the pancreas^11^ and the intestinal immune system^12^, as well as to play a role in diseases such as clear cell renal cell carcinoma^1,13,14^, our understanding of how *SETD2* heterozygosity culminates in LLS remains unclear. However, there have been some recent insights into the role of SETD2 in neural development. For instance, a recent study investigated how SETD2 regulates murine cortical development through the conditional ablation of this factor from the dorsal telencephalon^4^. Using an *Emx1*^*Cre*^*-*based approach, the authors selectively ablated *Setd2* from the mouse dorsal telencephalon. This led to disrupted areal patterning of the neocortex, impaired reciprocal cortico-thalamic connectivity and aberrant behavioural responses related to social interaction, motor learning and spatial memory in homozygous conditional knockouts. Conditional heterozygous knockouts did not exhibit these phenotypes. Furthermore, transcriptomic analyses of the homozygous mutant cortices revealed alterations to the *cPcdh* superfamily of cadherin molecules, among others^4^. The authors subsequently expanded upon these findings, revealing homozygous mutant mice also exhibited deficits in lamination, migration and maturity within the hippocampal cornu ammonis (CA) 1 region^15^. Collectively, these studies provide an important foundation for understanding how SETD2 regulates cortical development. However, there are several important outstanding issues regarding the phenotypes observed in this model of *Setd2*-deficiency within the mouse forebrain. Specifically, is the size of the adult brain outside the cortex affected in mutant mice, and what are the global connectivity changes within the neocortex and other regions of the brain that retain SETD2 expression? In addition, is the hippocampal dentate gyrus, a key structure that regulates learning and memory, affected in homozygous mutant mice? Here, we addressed these gaps, using mice carrying a conditional *Setd2* allele (*Setd2*^*fl/fl*^) crossed to mice carrying a codon-improved Cre recombinase driver under the control of the dorsal telencephalon-specific gene, *Emx1* (*Emx1*^*iCre/*+^)^16^. Using these *Setd2*^*fl/fl*^*; Emx1*^*iCre/*+^ knockout (cKO) mice, we revealed reduced volume for many areas of the adult brain, and alterations to the whole-brain connectome, including changes to the hippocampal connectome. We also highlight how *Setd2* ablation results in abnormal development of the adult dentate gyrus, including alterations to adult neural stem cell and immature neuronal populations. The findings shed further light on the role of SETD2 during brain development and may be informative regarding LLS.

## Results

### *Setd2* ablation from the dorsal telencephalon results in reduced volume of the adult brain

**Fig. 1:**
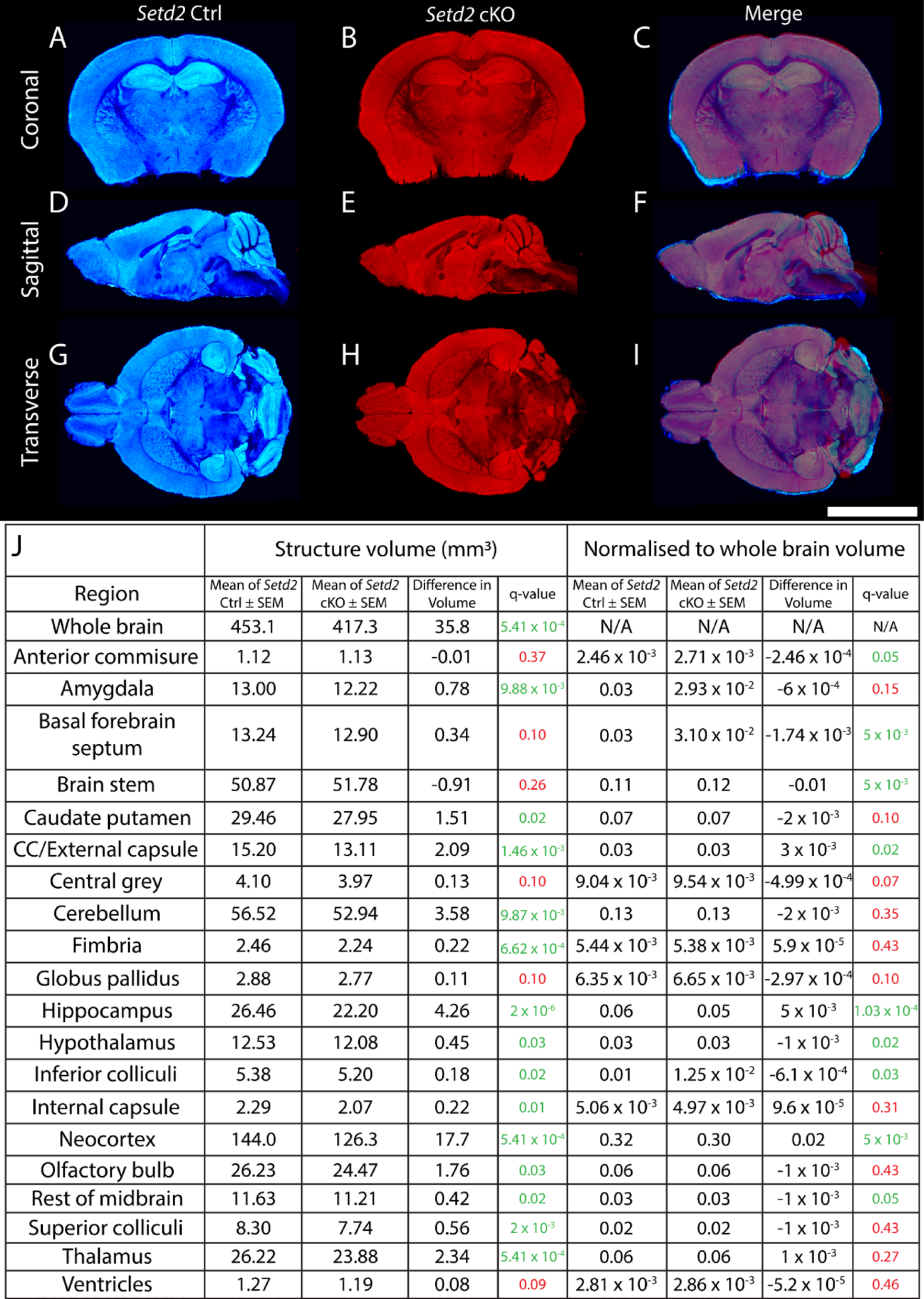
Volumetric analyses reveal that *Setd2* cKO brains are smaller. Volumetric model-based segmentation analyses of *Setd2* Ctrl (**A**, **D**, **G**) and cKO (**B**, **E**, **H**) brains were performed using the AMBMC whole brain atlas, and representative images of Ctrl and cKO brains were overlaid to show differences in volume (**C**, **F**, **I**). Volume of each structure, differences in volume, and normalised structural volume are shown in J. The corpus callosum, hippocampus, neocortex and thalamus, among other sub-substructures, were significantly reduced in absolute volume, as was the entire brain. Further delineation of structures and their associated volumes are shown in Supp. File 1. All data in J were analysed using multiple unpaired t-tests with a FDR of 5%. All data are represented as mean ± SEM. All values are rounded to 2 decimal places. Differences in (normalised) volume are represented as Ctrl – cKO. Scale bar represents 1 mm. CC = corpus callosum. For all analyses, n = 10 and n = 9 were used for Ctrl and cKO brains respectively.

Previous work has shown that conditional ablation of *Setd2* from the dorsal telencephalon culminates in a 10% smaller cortical width and hemispheric area in postnatal day (P)7 mice^4^. This prompted us to ask whether this phenotype was confined to the cortical hemispheres, or if the effects were more widespread. To investigate this, we used a similar model as Xu and colleagues^4^, namely a conditional *Setd2* allele^17^ crossed to an *Emx1*^*iCre/*+^ driver^18^, which should ensure more efficient ablation than the *Emx1*^*IRES Cre*^ line used previously^4^. In this model, *Setd2* is ablated from neural stem cells within the dorsal telencephalon from embryonic (E) day 10.5^19^. As such, most neurons and glia within the adult neocortex and hippocampus of Cre-expressing homozygous mice (barring interneurons, microglia and some oligodendrocytes) will lack SETD2 expression. To assess adult brain size, we performed volumetric magnetic resonance imaging (MRI) to compare brain volume in homozygous mice (*Setd2*^*fl/fl*^*; Emx1*^*iCre/*+^*— *hereafter called cKO mice) with controls (either *Setd2*^*fl/*+^*; Emx1*^+*/*+^ mice or *Setd2*^*fl/fl*^*; Emx1*^+*/*+^ mice). Analysis of these data confirmed a reduction in the size of the neocortex in cKO mice in comparison to controls, consistent with previous reports^4^ (Fig. 1A–J). Other areas derived from the dorsal telencephalon also exhibited reduced volume in the cKO, including the hippocampus and corpus callosum. These findings were also validated when performing haematoxylin and eosin staining of the adult brain across a range of different Bregma levels (Supp. Figure 1A–M). Critically, we also revealed that the whole adult cKO brain had a significantly reduced volume, and that areas within the diencephalon (thalamus, hypothalamus), cerebellum and brainstem (midbrain, inferior colliculi, superior colliculi) were also smaller in the cKO, despite being derived from neural stem cells that did not undergo *Setd2* ablation. These data suggest that the loss of *Setd2* from the dorsal telencephalon during development elicits phenotypes that extend beyond the neocortex.

These volumetric data were derived using a standard mouse brain atlas, which provides analyses of 20 broad regions of interest^20^. While this provides a solid basis for interrogating volumetric data, these atlases often lack the resolution necessary to elucidate changes in specific sub-regions of the brain. Additionally, previous studies into *Setd2-*deficiency in the neocortex have shown alterations of the size and location of discrete cortical regions^4^, emphasising that finer resolution atlases may reveal greater insights. Therefore, we partitioned our data, and analysed hippocampal^21^, diencephalic^22^ and neocortical^23^ regions in more detail, in order to determine which substructures within each of these areas were affected in the cKO. Within the hippocampus, all 15 substructures were found to be significantly reduced in the cKO in comparison to the control (Supp. File 1). Similarly, of the 94 diencephalic structures analysed, 38 structures showed significant reductions in volume in the cKO; these structures largely corresponded to different thalamic nuclei (Supp. File 1). Finally, of the 74 neocortical structures analysed, almost all (71/74) showed significant reductions in volume in the cKO (Supp File 1). Using an orthogonal approach (tensor-based morphometry), we validated these findings, showing broad alterations in volume within the brains of adult cKO mice (Supp. Figure 2A–I)^24–26^. Importantly, we also validated that analyses of regional volumes did not suffer from biases resulting from arealisation defects, as we were able to confirm that the software accurately mapped S1 onto the barrel cortex in the face of areal shifts using constrained spherical deconvolution mapping (Supp. Figure 3A–L). These findings suggest that there are significant reductions in cKO brain volume across specific regions within and outside of the dorsal telencephalon, including the neocortex, hippocampus, diencephalon, brainstem and cerebellum.

### *Setd2* cKO brains exhibit localised connectivity differences

**Fig. 2:**
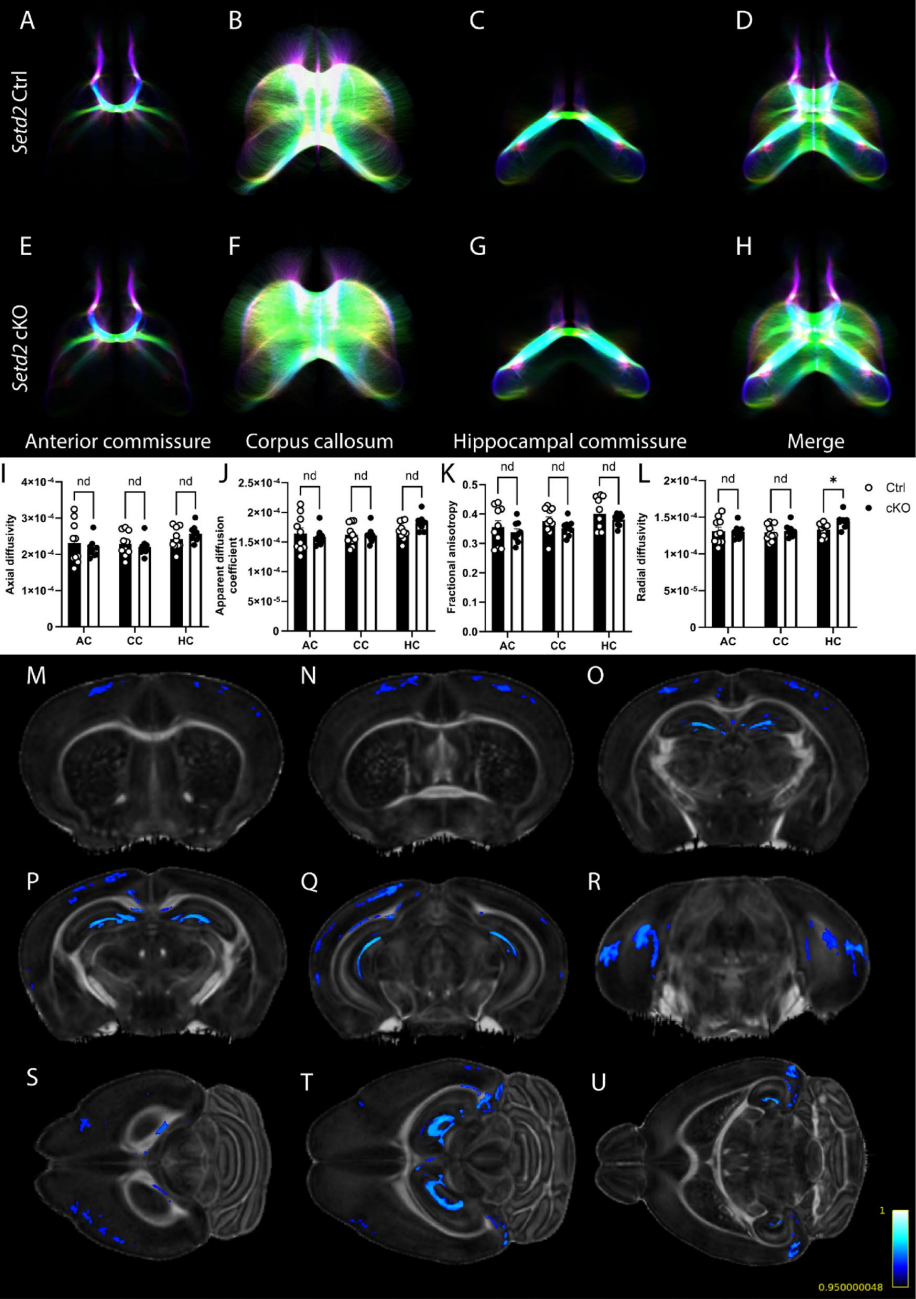
*Setd2* cKO mice largely display altered DTI metrics within the grey matter, but not the white matter, of regions connected by the major forebrain commissures. Representative tractography of *Setd2* Ctrl (**A**–**D**) and cKO (**E**–**H**) brains, highlighting the anterior commissure (AC; **A**, **E**), corpus callosum (CC; **B**, **F**) and hippocampal commissure (HC; **C**, **G**). All three tracts are represented as a merge panel (**D**, **H**). Diffusion tensor imaging (DTI) metrics (axial diffusivity (**I**), apparent diffusion coefficient (**J**), fractional anisotropy (**K**) and radial diffusivity (**L**)) are represented. No differences were noted in any of the major forebrain commissures, except for a significant increase in radial diffusivity within the hippocampal commissure (**L**). Voxel based morphometry (VBM) analyses of *Setd2* Ctrl and cKO are shown in representative average-brain templates for coronal (**M**–**R**) and transverse (**S**–**U**) planes. VBM revealed decreased (Ctrl > cKO) average fractional anisotropy (blue) within both white matter and grey matter substructures. Decreased FA is represented across the rostrocaudal axis (with M being most rostral, and R being most caudal), as well as along the dorsoventral axis (with S being most dorsal and U being most ventral). No increases in FA were detected using VBM analyses, in agreeance with other findings within this article. Colours for DTI represent the following directionality: green is left–right oriented, red is dorso-ventral oriented, blue is rostro-caudal oriented; oblique fibre orientations are a combination of these colours. nd = no difference; * *p* < 0.05. For all analyses, n = 10 and n = 9 were used for Ctrl and cKO brains respectively.

Xu and colleagues used anterograde AAV labelling to reveal altered reciprocal cortico-thalamic connectivity in *Setd2*^*fl/fl*^*;Emx1*^*IRESCre/*+^ mice^4^. To address if connectivity deficits are more widespread, we performed diffusion tensor magnetic resonance imaging (DTMRI), which allowed us to assess axon tract integrity and whole-brain connectivity. We first considered axonal integrity using DTMRI metrics, including fractional anisotropy (FA: this measures the degree of directionally dependent water diffusion in tissue), apparent diffusion coefficient (ADC: this measures the overall rate of microscopic water diffusion), axial diffusivity (AD: this measures parallel microscopic water diffusion) and radial diffusivity (RD: this measures perpendicular microscopic water diffusion)^27^. Interestingly, despite the reported alterations in cortico-thalamic connectivity in cKO mice^4^, seeding regions of interest (ROIs) within the diencephalon and basal ganglia, such as thalamic nuclei and the caudate putamen, and the primary somatosensory or motor cortices, did not reveal any changes in these DTMRI metrics (Supp. Figures 4A–P and 5A–P). These data suggest that, while axonal connectivity between the cortex and thalamus may be aberrant^4^, and the volume of the internal capsule was reduced in the cKO (Fig. 1J), the microstructural integrity of the axons linking these structures was not affected. Likewise, FA, ADC, AD and RD measurements of axons within the major forebrain commissures–corpus callosum, hippocampal commissure and anterior commissure–were comparable between cKO and control mice (Fig. 2A–K), apart from RD within the hippocampal commissure, which was significantly increased in the cKO (Fig. 2L). We further confirmed this through the use of an orthogonal approach (voxel-based morphometry (VBM)), whereby each voxel was analysed within the grey and white matter individually^26^. In doing so, we confirmed no significant alterations to FA within the major forebrain commissures but did find significant reductions in FA within the motor and somatosensory cortices of cKO brains (Fig. 2M–U). These findings may suggest changes to FA within the grey matter of affected regions (such as S1)^4^ which may not be seen using standard DTI assessments.

**Fig. 3:**
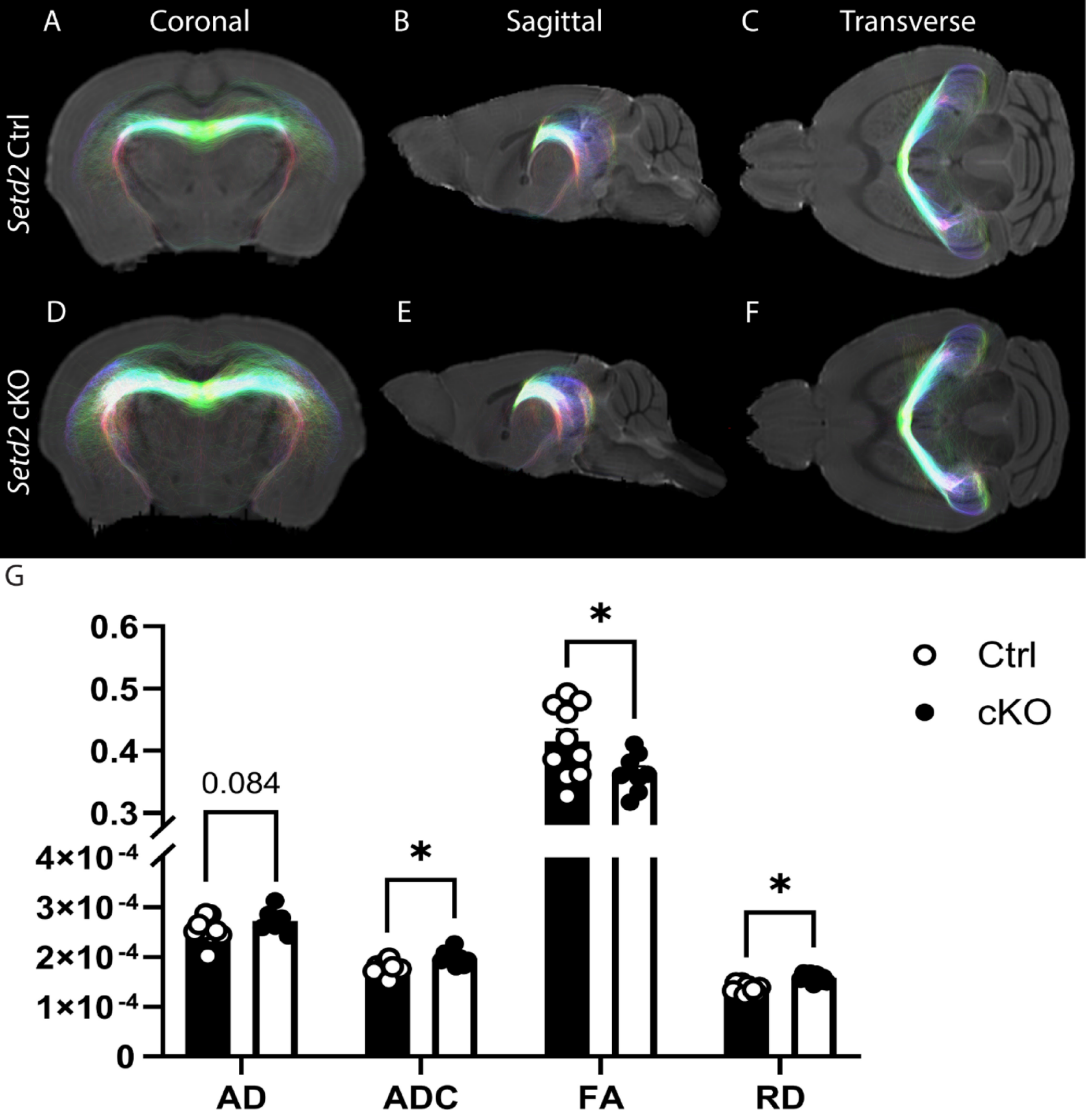
*Setd2* cKO mice exhibit abnormal bilateral hippocampal connectivity. Representative tractography of *Setd2* Ctrl (**A**–**C**) and cKO (**D**–**F**) brains in coronal (**A**, **D**), sagittal (**B**, **E**) and transverse (**C**, **F**) planes. All tracts presented represent connectivity between the left hippocampal body (HpL) and the right hippocampal body (HpR), with only tracts passing through the fornix (For) being shown. Diffusion tensor imaging (DTI) metrics (axial diffusivity (AD), apparent diffusion coefficient (ADC), fractional anisotropy (FA) and radial diffusivity (RD)) are represented (**G**). All metrics but AD were significantly altered, with significant increases being seen in ADC and RD and significant decreases in FA. Colours for DTI represent the following directionality: green is left–right oriented, red is dorso-ventral oriented, blue is rostro-caudal oriented; oblique fibre orientations are a combination of these colours. * *p* < 0.05. For all analyses, n = 10 and n = 9 were used for Ctrl and cKO brains respectively.

Given that radial diffusivity was increased in the hippocampal commissure (Fig. 2L), and the hippocampus receives afferent fibres from numerous other regions (including the entorhinal cortex, prefrontal cortex and the anterior cingulate gyrus)^28^ which were reduced in size (Supp. File. 1), we next wanted to investigate the hippocampal formation in greater detail. To this end, we isolated ROIs of each hippocampal body (HpL and HpR) and analysed tracts passing between these bodies via the fornix (For) via tractography. Analyses of this “circuit” revealed significantly increased ADC and RD, in line with the HC increases (Fig. 2L) and significantly decreased FA (Fig. 3A–G). These data are consistent with the reduced size of the hippocampus and suggest that this part of the brain is particularly affected by loss of SETD2 activity, as has been previously shown^15^. Indeed, using VBM, we confirmed that there were decreases to FA within the hippocampal formation, in line with what we saw using DTI (Fig. 2M–U). Altogether, these findings are suggestive of region-specific differences in the microstructural integrity of connections within cKO brains.

### *Setd2* cKO brains exhibit altered whole-brain structural connectivity

**Fig. 4:**
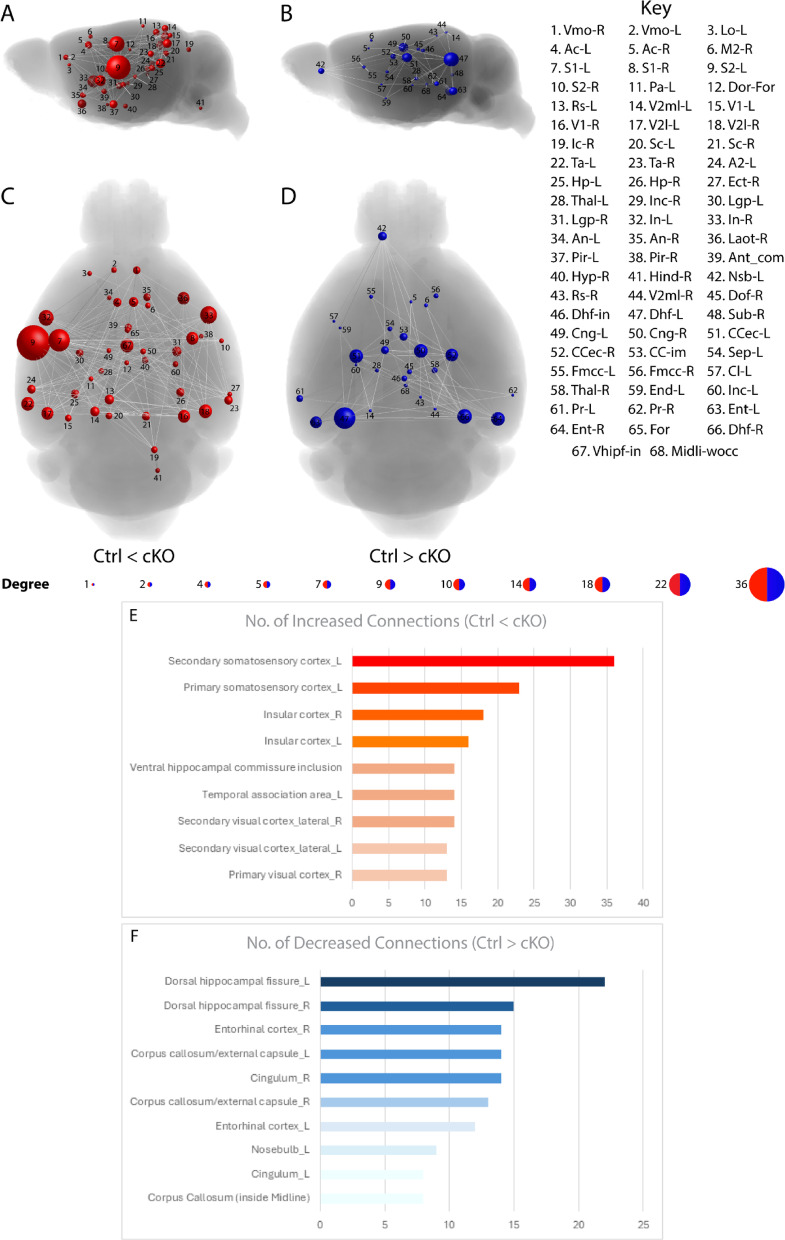
Structural connectome mapping reveals abnormal inter-nodal connectivity within *Setd2* cKO brains. Connection-wise comparisons between *Setd2* controls compared to cKO brains was performed using Network Based Statistic analysis. The intensity component (total number of connections) and primary threshold (*t* = 2.8, *p* = 0.0064) results were used for all representations above. Brain nodes are represented as circles scaled in size according to the degree of change in a node, which corresponds to the number of different connections made with that node. Abnormal nodes are shown in sagittal (**A**, **B**) and axial (**C**, **D**) views. Nodes that had increased connectivity in *Setd2* cKO mice compared to controls are depicted in red, while nodes with decreased connectivity are depicted in blue. (**A**, **C**) Compared to control brains, *Setd2* cKO brains predominantly show a significantly increased degree of inter-nodal connections within primary integration areas, as represented in the bar graph provided (**E**). However, *Setd2* cKO brains primarily show a significantly decreased degree of inter-nodal connectivity within hippocampal structures, as well as structures for long range projections, as represented in the bar graph provided (**F**). For all analyses, n = 10 and n = 9 were used for Ctrl and cKO brains respectively. A2-L, Secondary auditory cortex, Left; Ac-L, Anterior cingulate, Left; Ac-R, Anterior cingulate, Right; An-L, Accumbens nucleus, Left; An-R, Accumbens nucleus, Right; Ant_com, Anterior commissure; CC-im, Corpus callosum (inside midline); CCec-L, Corpus callosum/external capsule, Left; CCec-R, Corpus callosum/external capsule, Right; Cl-L, Claustrum, Left; Cng-L, Cingulum, Left; Cng-R, Cingulum, Right; Dhf-in, Ventral hippocampal commissure inclusion; Dhf-L, Dorsal hippocampal fissure, Left; Dhf-R, Dorsal hippocampal fissure, Right; Dof-R, Dorsal fornix, Right; Dor_for, Dorsal Fornix; Ect-R, Ectorhinal cortex, Right; End-L, Endopiriform nucleus, Left; Ent-L, Entorhinal cortex, Left; Ent-R, Entorhinal cortex, Right; Fmcc-L, Forceps minor of the corpus callosum, Left; Fmcc-R, Forceps minor of the corpus callosum, Right; For, Fornix; Hind-R, Hindbrain, Right; Hp-L, Hippocampus, Left; Hp-R, Hippocampus, Right; Hyp-R, Hypothalamus, Right; Ic-R, Inferior colliculus, Right; In-L, Insular cortex, Left; In-R, Insular cortex, Right; Inc-L, Internal capsule, Left; Inc-R, Internal capsule, Right; Laot-R, Lateral olfactory tract, Right; Lgp-L, Lateral globus pallidus, Left; Lgp-R, Lateral globus pallidus, Right; Lo-L, Lateral orbital cortex, Left; M2-R, Secondary motor cortex, Right; Midli-wocc, Midline (except corpus callosum); Nsb-L, Nosebulb, Left; Pa-L, Parietal cortex, Left; Pir-L, Piriform cortex, Left; Pir-R, Piriform cortex, Right; Pr-L, Perirhinal cortex, Left; Pr-R, Perirhinal cortex, Right; Rs-L, Retrosplenial cortex, Left; Rs-R, Retrosplenial cortex, Right; S1-L, Primary somatosensory cortex, Left; S1-R, Primary somatosensory cortex, Right; S2-L, Secondary somatosensory cortex, Left; S2-R, Secondary somatosensory cortex, Right; Sc-L, Superior colliculus, Left; Sc-R, Superior colliculus, Right; Sep-L, Septum, Left; Sub-R, Subiculum, Right; Ta-L, Temporal association area, Left; Ta-R, Temporal association area, Right; Thal-L, Thalamus, Left; Thal-R, Thalamus, Right; V1-L, Primary visual cortex, Left; V1-R, Primary visual cortex, Right; V2l-L, Secondary visual cortex, lateral part, Left; V2l-L, Secondary visual cortex, lateral part, Right; V2ml-L, Secondary visual cortex, mediolateral part, Left; V2ml-R, Secondary visual cortex, mediolateral part, Right; Vhipf-in, Ventral hippocampal commissure inclusion; Vmo-L, Ventromedial orbital cortex, Left; Vmo-R, Ventromedial orbital cortex, Right.

In addition to assessing axonal integrity, DTMRI can also be used to investigate global structural connectivity within the brain. Previous studies investigating ASD in patients have utilised network-based statistic (NBS) approaches to model how patients may present with altered functional connectivity^29^. The authors found altered connectivity using resting state functional MRI and showed alterations to regions involved in memory and learning (hippocampus), as well as socialisation and emotional responses (the limbic system). Based upon these findings, we theorised that the use of a similar NBS approach would allow us to determine whether there were alterations to whole-brain connectivity in cKO mice. To do this, we utilised the NBS toolbox^30^, allowing us to visualise structural connectivity, and perform connection-wise comparisons between “nodes” (regions) of the brain^31^. Using a 106-node template, we constructed the structural connectome to determine which nodes showed altered (increased or decreased) connectivity in cKO brains compared to controls (Fig. 4A–D). NBS analyses of control and cKO brains revealed increased connectivity primarily within areas associated with sensory processing, such as the somatosensory and visual cortices (Fig. 4E), and decreased connectivity primarily within areas associated with the hippocampus i.e., dorsal hippocampal fissures, as well as projections and connectivity i.e., CC and cingulum (Fig. 4F; see Supp. File 1 for all findings). LLS, which is caused by heterozygous loss of function of *SETD2*, is often comorbid with ASD and language acquisition delays. In line with this, we observed alterations across the brain in regions and “networks” typically associated with ASD aetiology using a structural NBS approach^29^, suggesting that ablation of *Setd2* from the neocortex results in altered structural connectivity within the adult mouse brain.

### *Setd2* ablation results in abnormal cellular composition of the adult hippocampus

**Fig. 5:**
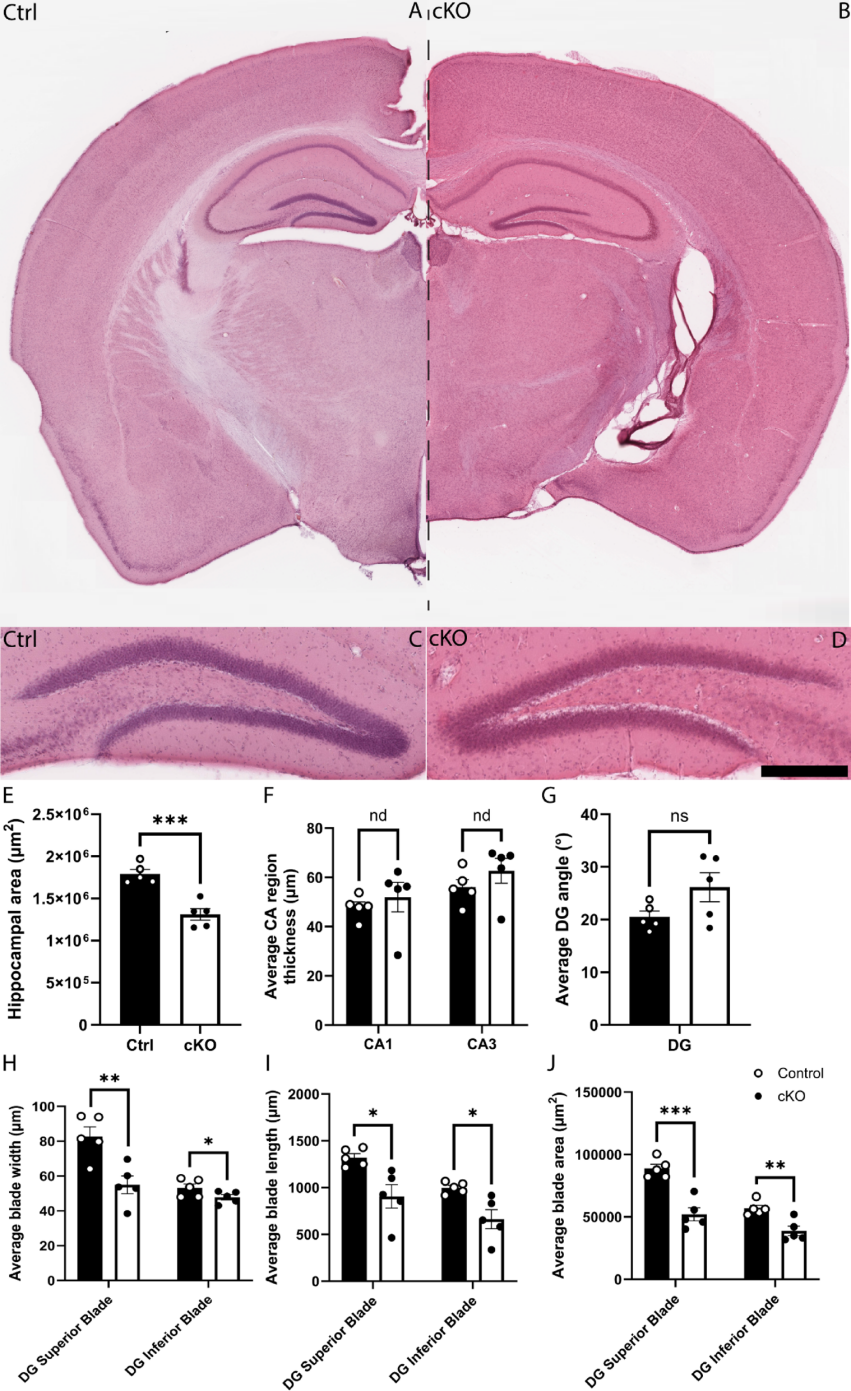
*Setd2* cKO brains show reductions in size across the adult hippocampus. (**A**) Coronal sections taken at the level of the dentate gyrus of the hippocampus from *Setd2* control (**A**, left) and cKO (**B**, right) brains, stained using haematoxylin and eosin. Higher magnification zooms of the dentate gyrus are shown below (**C**, **D**). Quantification of (**E**) average total hippocampal area, (**F**) average hippocampal CA region thickness, (**G**) average angle between blades of the DG (at the hilus), (**H**) average DG blade width, (**I**) average DG blade length and (**G**) average DG blade area. nd = no difference, ns = not significant, * *p* < 0.05; ** *p* < 0.01; *** *p* < 0.001. Scale bar in D represents 1 mm for A & B, and 250 μm for C & D. CA = cornu ammonis, DG = dentate gyrus, IB = inferior blade, SB = superior blade. For all analyses, an n = 5 was used for each genotype.

**Fig. 6:**
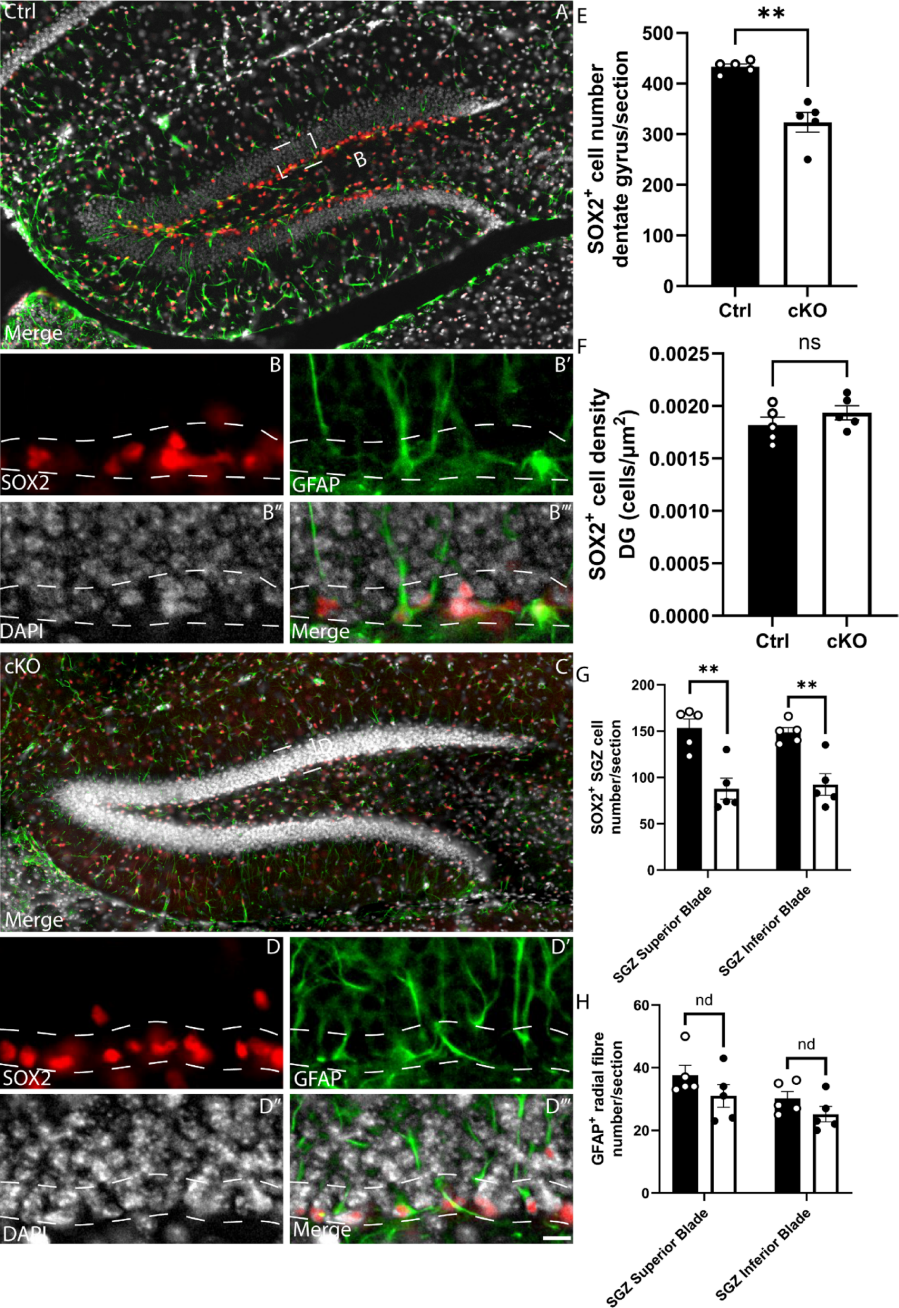
*Setd2* cKO brains exhibit alterations in cellular populations within the hippocampus. Coronal sections taken at the level of the dentate gyrus of the hippocampus from *Setd2* control (**A**–**B'"**) and cKO (**F**–**D'"**) brains, revealing the expression of SOX2 (red), GFAP (green) and DAPI (white). Quantification of (**E**) the average number of SOX2^+^ cells within the DG, (**F**) density of those cells in the DG, (**G**) average number of SOX2^+^ cells within the SGZ and H) average number of radially oriented GFAP^+^ fibres. nd = no difference, ns = not significant; ** *p* < 0.01. Scale bar in **D'"H** represents 25 µm for all large panels, and 5 µm for all insets. For all analyses, an n = 5 was used for each genotype.

Previous studies in cKO mice have shown arealisation defects in the cortex, albeit with typical cortical lamination^4^, as well as lamination and migratory deficits within CA1 of the hippocampus^15^. Additionally, Xu and colleagues identified abnormal behavioural and learning responses^4^. Because of the important role played by the dentate gyrus (DG) in these processes, we next turned our attention to analysing the DG. We first looked at gross morphology of the adult control and cKO brain using haematoxylin and eosin staining. The area of the hippocampus was significantly reduced, and the dentate gyrus blades were smaller in area, thickness and length (Fig. 5A–J), consistent with our MRI analyses (Fig. 1J). Both excessive cell death and reduced proliferation can contribute to changes in structure size, however, using the expression of the apoptotic marker CC3^32^, we did not observe any significant changes in apoptosis within the DG of cKO mice in comparison to controls (Supp. Figure 6A–E). The adult mouse hippocampus is one of the few regions of the forebrain in which neurogenesis continues throughout life, with quiescent and proliferative adult neural stem cells (aNSCs) present within the subgranular zone (SGZ) of the dentate gyrus^33^. Could impaired adult neurogenesis contribute to the reduction in hippocampal volume? To address this, we assessed the expression of SOX2 (a marker for aNSCs and astrocytes more broadly)^34^ and GFAP (an astrocytic/aNSC marker)^35^. Cell counts revealed fewer SOX2^+^ cells within the dentate gyrus of the cKO (Fig. 6A–F), and, importantly, a reduced number of SOX2-expressing cells within the SGZ of the mutant (Fig. 6G), suggestive of reduced aNSCs in the cKO. Despite this, the number of radially orientated GFAP-expressing fibres within the granular zone of the cKO was not significantly different in the cKO compared to controls (Fig. 6H), which may point to differences in the numbers of quiescent versus active aNSCs within the SGZ^36^.

**Fig. 7:**
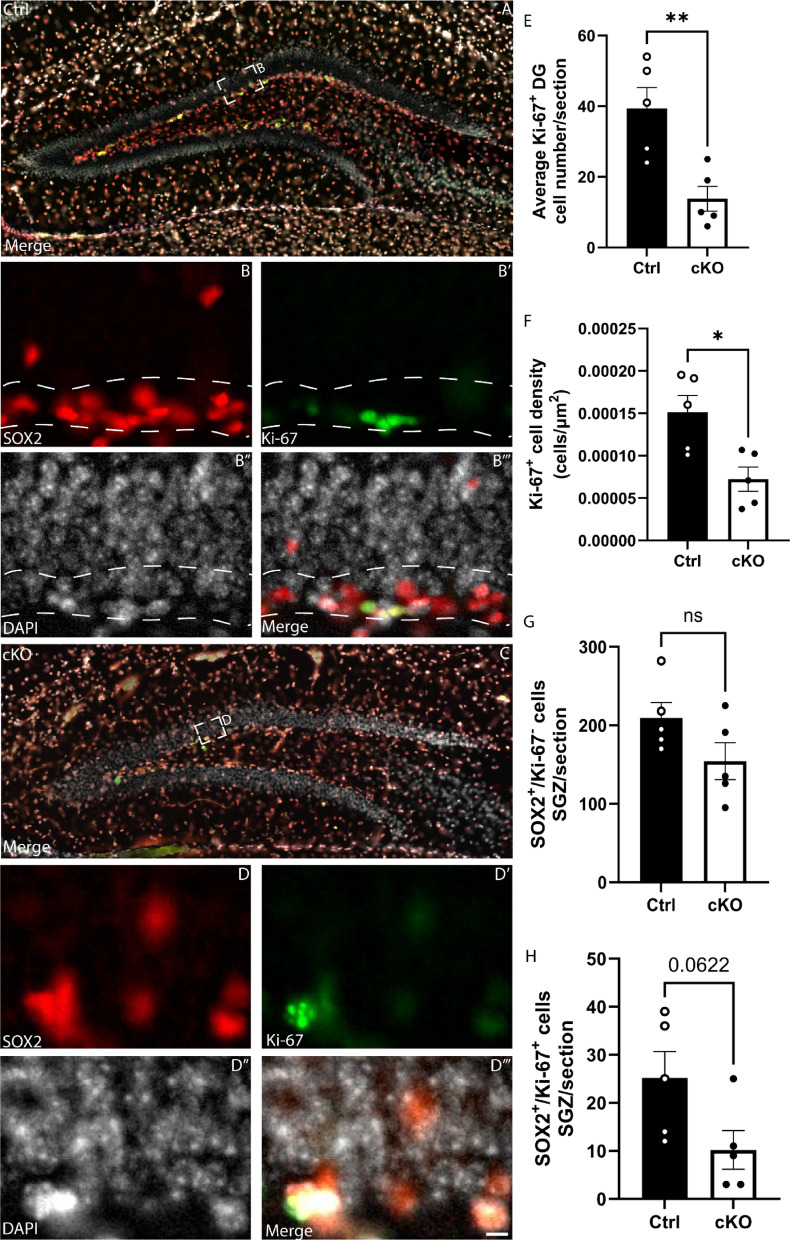
Proliferation within the SGZ is affected in *Setd2* cKO brains. Coronal sections taken at the level of the dentate gyrus of the hippocampus from *Setd2* control (**A**–**B’”**) and cKO (**C**–**D’”**) brains, revealing the expression of SOX2 (red), Ki67 (green) and DAPI (white). Quantifications of (**E**) the average number of KI-67^+^ cells in the SGZ, (**F**) the average density of KI-67^+^ cells in the SGZ, G) the average number of SOX2^+^/KI-67^-^ cells in the SGZ and (**H**) the average number of SOX2^+^/KI-67^+^ cells in the SGZ. Scale bar in **D’”** represents 25 µm for all large panels, and 5 µm for all insets. ns = not significant, * = *p* < 0.05, ** = *p* < 0.01. For all analyses, an n = 5 was used for each genotype.

**Fig. 8:**
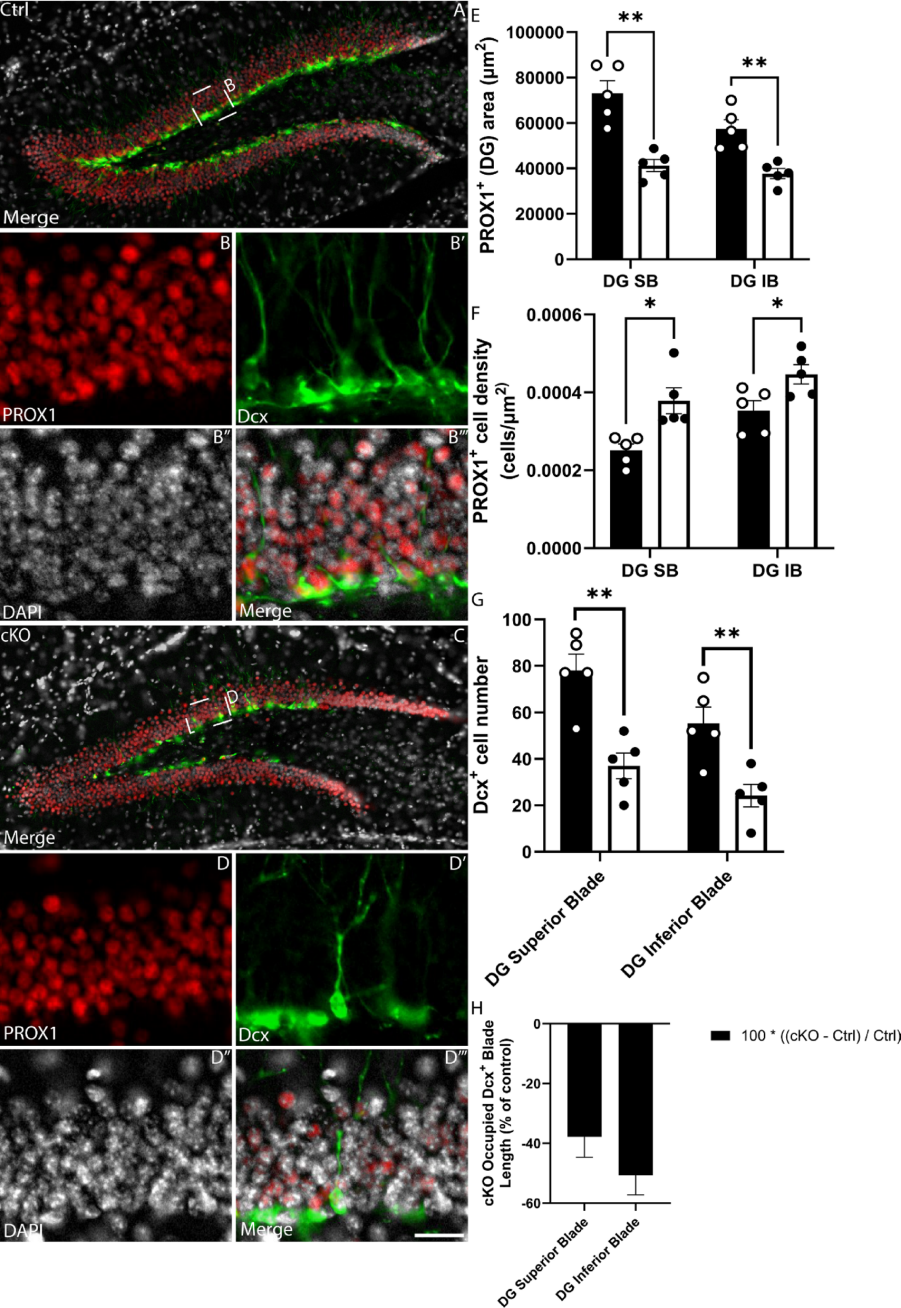
*Setd2* cKO brains show alterations in hippocampal neuronal populations. Coronal sections taken at the level of the dentate gyrus of the hippocampus from *Setd2* control (**A**–**B’”**) and cKO (**C**–**D’”**) brains, revealing the expression of PROX1 (red), Dcx (green) and DAPI (white). Quantification of (**E**) the PROX1^+^ area of each blade of the dentate gyrus, (**F**) the density of PROX1 cells within the DG, (**G**) the number of Dcx^+^ neuroblasts and (**H**) the length of each blade of the cKO DG occupied by Dcx^+^ neuroblasts as a percentage of control brains. ns = not significant; * *p* < 0.05; ** *p* < 0.01; *** *p* < 0.001. Scale bar in **D’”** represents 100 µm for all large panels, and 20 µm for all insets. For all analyses, an n = 5 was used for each genotype.

To probe this further, we next aimed to determine if there was a reduction in the number of proliferating aNSCs. To assess proliferation within the hippocampal dentate gyrus directly, we performed immunofluorescence staining using antibodies against SOX2 and KI-67, a marker of cells progressing through the cell cycle. Cell counts revealed a significant reduction in the number and density of KI-67-expressing cells within the dentate gyrus of the cKO, as well as a small, though non-significant reduction in the number of cells within the SGZ expressing both SOX2 and KI-67 (proliferating aNSCs; *p* = 0.0622; Fig. 7A–H). Expression of PROX1 (a marker for mature granule neurons) revealed a reduction in the granular zone of the superior and inferior blades of the dentate gyrus (Fig. 8A–E), which was also accompanied by reduced number, density and blade length occupied by immature neurons (Doublecortin (DCX)-positive cells; Fig. 8G–H). Taken together, these findings suggest that *Setd2* ablation results in a reduction in the number of active aNSCs, accompanied by a reduction in newborn neurons in the adult brain.

Finally, as previous studies did not consider how ablation of *Setd2* from the dorsal telencephalon affects glial populations^4,15^, we investigated this feature within the hippocampus and neocortex. As outlined above, we identified a decrease in the total number of SOX2-expressing cells within the entire dentate gyrus (Fig. 6E), suggestive of a reduction in astrocyte numbers within the hilar region of the dentate gyrus. Similarly, analysis of OLIG2 expression revealed a significant reduction in the total number of cells of the oligodendrocyte lineage in the cKO hippocampus (Supp. Figure 7A–C). However, we did not observe any difference in the number or density of SOX2- or OLIG2-expressing cells within the motor cortex (Supp. Figure 8A–P).

## Discussion

In the current study, we have uncovered previously unappreciated roles for SETD2 in regulating cortical development. We reveal that SETD2 regulates brain volume and whole-brain structural connectivity, while at the circuit level *Setd2* ablation results in deficits within hippocampal tract integrity. We highlight how *Setd2* ablation results in reduced proliferation of aNSCs within the subgranular zone of the dentate gyrus, resulting in reduced neurogenesis as well as alterations to several different cellular populations within the adult hippocampus. Altogether, these findings expand upon previously established phenotypes shown to exist within the cKO brain^4,15^, and highlight a key role for SETD2 in neural development.

Within this study, we have highlighted reductions in brain size and volume in *Setd2* cKO mice. Previous studies into mouse models of other epigenetic genes have shown brain size changes depending on gene dosage. These include models of *Dnmt3a* deficiency, heterozygosity of which causes the human overgrowth disorder Tatton-Brown-Rahman Syndrome, which manifest with tibial/femoral length increase, adult onset obesity and brain size alterations^37,38^. Studies of three different models of *Dnmt3a* deficiency revealed that despite craniofacial morphometry being largely unchanged and no change in skull size being noted^37,38^, there was a late-adulthood onset of reduced brain volume, not noted at 8 weeks of age, which was worsened by mutation severity (R878H vs P900L mutations)^37^. Yet another study into *Chd8*, a DNA binding protein implicated in chromatin remodelling, autism spectrum disorder and CHD8-related overgrowth disorder^39–42^, revealed how gene dosage drives distinct outcomes for brain growth^43^. While haploinsufficiency for *Chd8* resulted in brain overgrowth, severe reductions in gene expression (~ 91% reduction at protein level) resulted in brain undergrowth^43^. Studies of *Dnmt3a* and *Chd8* show similarities to the findings we and others^4,15^ have observed in *Setd2* cKO mice, and suggest that total ablation of *Setd2* may result in different outcomes as opposed to heterozygous ablation—what then is driving these brain size reductions in our *Setd2*-deficient mice?

There are likely numerous distinct mechanisms at work which are driving the reductions in brain size. Previous studies into regulators of hippocampal volume have revealed that reduced neurogenesis, as well as stress, drives CA3/DG-specific reductions in volume^44^. Chronic stress reduced volume across the entire hippocampus, while inhibition of neurogenesis reduced volume only within the dentate gyrus (after 4 weeks), and then the CA3 region (after 16 weeks)^44^. Because *Setd2* cKO mice have previously been demonstrated to exhibit anxiety-like phenotypes^4^, and we have shown here that *Setd2* ablation results in reduced neurogenesis, we hypothesise that hippocampal volume reductions result from a combination of reduced neurogenesis and an increased anxiolytic effect. As for volume within the cortex, one possibility for reduced neocortical volume may lie in the connectivity deficits we and others have characterised^4^. Reduced volume and connectivity, as well as aberrant targeting of connections^4^, have previously been linked to reduced brain volumes^18,31^—indeed, a previous study^18^ where *Emx1* was used to ablate the gene-of-interest revealed reduced volumes in regions which do not derive from *Emx1*-expressing progenitors, similar to our findings herein. Against this backdrop, we posit that the volumetric reductions in the hippocampus derive from neurogenesis-related mechanisms, and neocortical reductions derive from abnormal connectivity.

Previous studies^4^ have shown that cKO brains show altered reciprocal corticothalamic connectivity. In the current study, we were unable to directly assess this possibility using DTI techniques but have highlighted that axons within these “circuits” do not show altered DTI metrics (i.e. changes to FA), while axons within the hippocampal “circuit” are affected in their axonal microstructural integrity. One possibility for these differences likely comes down to glial, and specifically oligodendrocyte, populations. While previous studies have failed to consider these crucial populations^4,15^, here we have shown a reduction in the number of hippocampal glial populations. Oligodendrocytes are crucial in myelinating axons throughout the brain, and myelination status could explain the alterations seen in DTI metrics in our study. While we were unable to directly assess myelination differences in this study, myelination has been implicated in neurodevelopmental disorders such as autism previously^45^, and may explain differences seen within cKO brains in relation to both DTI metrics and altered connectivity. As such, we suggest that oligodendrocyte differences may potentially contribute to hippocampal “circuit” alterations, while altered connectivity may have another underlying cause yet to be identified.

In the current work, we have focused our analyses on adult mice. However, since *Setd2* is ablated from ~ E10.5 through *Emx1*^19^, it is likely that these phenotypes originate much earlier in development. Indeed, Xu and colleagues identified reduced brain hemispheric area as early as P7^4^, suggesting that the onset of brain volume reductions may be occurring earlier in development. In future studies it would be important to conduct DT-MRI analyses at earlier postnatal, and indeed embryonic ages, to map the onset of deficits. Given the early onset of reduced brain size (P7) and the fact that major aspects of mouse brain connectivity are not complete until 2–3 weeks of age, with full maturation occurring even beyond this point^46,47^, what may be driving this early phenotypic onset? While Xu and colleagues have shown that apoptosis is not increased at P0, this does not preclude a later onset of increased cell death, or indeed other modalities of cell death^48,49^, driving reduced brain volume^4^. Assessment of cell death using complementary methods, such as TUNEL staining, across several ages may reveal if postnatal cell death is contributing to reduced brain size. Similarly, while proliferation has been assessed through the use of BrdU, Tbr2 and KI-67 stains^4,15^ and has shown typical cell proliferation across most of the embryonic brain, with the exception of elevated hippocampal neuroepithelium-localised KI-67^+^ cells at E15.5^15^, it is nevertheless possible that proliferation is reduced during the early postnatal period. This possibility could be assessed through the use of neurosphere assays to assess proliferation potential of neural stem cells.

Finally, we have demonstrated that *Setd2* ablation results in alterations to adult neural stem cell populations within the subgranular zone (SGZ) of the hippocampus, and that these cells are incompetent at producing newborn neurons. Adult neurogenesis is crucial for several reasons, including replenishment of dead/dying cells and plasticity for memory and learning^33,50,51^. Neurogenesis is also acutely linked to health and disease, including Alzheimer’s, Parkinsons and typical aging^50,52^. We have revealed that in the absence of *Setd2,* there are reductions in the number of active aNSCs residing in the SGZ, as well as the number of newborn neurons of the DG, suggesting that the active aNSCs that do exist are unable to produce the same numbers of newborn neurons in the absence of *Setd2*. Proliferative capacity of active aNSCs could be investigated using methods such as neurosphere assays to assess proliferative competency and stemness^53^. In addition, the use of methods such as single nucleus multiome sequencing methods (snRNA/snATACseq)^54^, combined with methods such as CUT&RUN for profiling of histone modifications^55^, or even spatial sequencing methodologies^56,57^, would allow for an in-depth characterisation of how these aNSCs differ in the absence of *Setd2*. Indeed, these sequencing methodologies would likely allow us to elucidate specific gene pathways or networks that may altered in the absence of *Setd2* and provide possible targets for future research. In closing, we have provided further characterisation of the phenotypes present in the cortices of *Setd2* cKO mice, highlighting previously unappreciated roles for SETD2 in neural development. Further research should be undertaken in more clinically relevant settings, such as the use of heterozygous murine models of LLS or in vitro approaches derived from patients own tissues, to continue to advance our understanding of the breadth of roles that SETD2 plays.

## Materials and methods

### Animals

Animals used in this study were bred at The University of Queensland under approval from the Institutional Animal Ethics Committee (2023/AE000007). To study the role of SETD2 in cortical development, a conditional allele was used; *Setd2*^*fl/fl*^, which was generated from embryonic stem cells as previously described^58^. This conditional line was crossed with mice carrying a codon-improved Cre recombinase under control of the *Emx1* promoter (*Emx1*^*iCre/*+^)^16^, which has been used successfully previously by our lab^18^ and others^59^. Use of this *Emx1*^*iCre/*+^ line enables the ablation of *Setd2* from neural stem cells of the dorsal telencephalon from approximately E10.5. This generated *Setd2*^*fl/*+^*; Emx1*^*iCre/*+^ progeny. These animals were crossed with *Setd2*^*flfl*^ mice to generate control (*Setd2*^*fl/fl*^; *Emx1*^+*/*+^ or *Setd2*^*fl/*+^; *Emx1*^+*/*+^; hereafter referred to as controls) or homozygous (*Setd2*^*fl/fl*^*; Emx1*^*iCre/*+^*;* hereafter referred to as cKO mice) animals. All experiments were performed according to the Australian Code of Practice for the Care and Use of Animals for Scientific Purposes and according to the ARRIVE guidelines. Pregnant females were acquired by placing male and female mice together overnight. The next day, females were inspected for the presence of a vaginal plug. The day of birth was designated as postnatal day (P) 0. Mice were housed in Optimice IVC caging, with double HEPA filter and built in ventilation. Food and water were available ad libitum. Animals at P69–P72 were used in this study and were collectively termed “adult” mice. Mice were genotyped by PCR; primers are available on request (Supp. Table 1). The experiments performed within this manuscript were performed on the same cohort of mice (i.e. the same mice used for MRI analyses were subsequently used for histological and immunohistochemical analyses).

### Magnetic resonance imaging

For volumetric and diffusion MRI analyses, adult *Setd2* control (male = 6, female = 4) and *Setd2* cKO (male = 6, female = 3) brains were used. Mice were deeply anesthetised with Lethabarb (1:50 dilution, 0.7–0.9 mL injection; VIRBAC PTY) and euthanised via transcardial perfusion with 0.1 M phosphate-buffered saline (PBS), followed by 4% (*w*/*v*) paraformaldehyde (pH 7; Sigma-Aldrich) in PBS. Before MRI scanning, the brains were washed in 0.1 M PBS with 0.2% *v*/*v* gadopentetate dimeglumine (Magnevist, Bayer, Leverkusen) for four days^60^. MRI data were acquired using a 16.4 T vertical bore microimaging system (Bruker Biospin, Rheinstetten; ParaVision v6.01) equipped with a Micro2.5 imaging gradient and a 15 mm linear surface acoustic wave coil (M2M, Brisbane, Australia). Three-dimensional (3D) T1/T2*-weighted FLASH structural images were acquired using a gradient echo imaging sequence with the following parameters: repetition time (TR) = 50 ms, echo time (TE) = 12 ms, bandwidth = 50 kHz, field of view (FOV) = 19.6 × 11.4 × 8.4 mm and matrix size = 654 × 380 × 280, which results in 30 μm isotropic image resolution, with an acquisition time of 30 min. 3D diffusion-weighted images (DWI) data were acquired using a Stejskal-Tanner DWI spin-echo sequence with TR = 200 ms, TE = 23 ms, δ/Δ = 2.5/12 ms, bandwidth = 50 kHz, FOV = 19.6 × 11.4 × 8.4 mm and matrix size = 196 × 114 × 84, image resolution = 100 μm, 30 direction diffusion encoding with b-value = 5000 s/mm2, two b = 0 images, with an acquisition time of 17 h. DWI datasets were zero-filled by a factor of 1.5 in all dimensions prior to Fourier transform to improve fibre tracking^61^.

To perform volumetric analyses, the Australian Mouse Brain Mapping Consortium atlases for whole brain (https://imaging.org.au/AMBMC/Model/), hippocampus (https://imaging.org.au/AMBMC/Hippocampus/), neocortex (https://imaging.org.au/AMBMC/Cortex/) and diencephalon (https://imaging.org.au/AMBMC/Diencephalon/) were registered to the FLASH images using FMRIB Software Library’s linear registration (FLIRT, fsl.fmrib.ox.ac.uk), followed by ANTs diffeomorphic registration (https://github.com/ANTsX/ANTs)^62^. Model-based segmentation of each atlases brain regions was performed on each sample and their volumes were measured using ITK-SNAP (https://www.itksnap.org/pmwiki/pmwiki.php)^63^. For all subsequent analyses, only the whole brain model atlas was used unless specified. To analyse the diffusion MRI data, image intensity were first bias corrected using ANTs N4BiasFieldCorrection and processed using MRtrix3 software (https://www.mrtrix.org/)^64^. Fiber orientation distribution (FOD) was reconstructed using constrained spherical deconvolution (CSD) method, and probabilistic tractography was performed using iFOD2 algorithm. Tractography was performed for specific major white matter tracts and ROIs, and for the whole brain for the structural connectome analyses. Firstly, the seeding regions of interest (ROIs) were manually drawn in the midsagittal and coronal sections of the colour vector map, and fibre tracks were generated for the corpus callosum, hippocampal commissure, and anterior commissure at 100 seeds per voxel. From these structures, parametric maps, including tract density imaging (TDI, which measures number of tracts within a voxel unit)^65^ and diffusion-tensor MRI (DTMRI) metrics, including fractional anisotropy (which measures the microstructural integrity of axonal tracts), and mean, radial and axial diffusivities (which measures the overall water motility, and in the perpendicular and parallel orientations to axonal bundles, respectively)^66^ were calculated for each structure and compared between control and *Setd2* cKO brains. For non-forebrain commissure tractographic analyses (hippocampus and M1/S1 to thalamus), ROIs taken from the 106 node Centre for Advanced Imaging (CAI)-John Hopkins MRI atlas^67^ were overlaid on brains and used to guide tractography. Voxel based morphometry was performed by firstly creating a multivariate template image using the ANTs program and followed by FSL Randomise with 1000 permutations and threshold-free cluster enhancement (FWE-corrected, *p* < 0.05). To perform structural connectome analyses, whole-brain probabilistic tractography was generated using 10 seeds per voxel. The modified Centre for Advanced Imaging (CAI)-John Hopkins MRI atlas^67^ was used to segment the whole brain tractography into a connectivity matrix comprising of 106 nodes. The degree of a node in a network corresponds to the number of connections a node has with other nodes. The Network Based Statistic (NBS) toolbox^30^ was used to detect changes in the brain connectivity network between control and *Setd2* cKO mouse brains. NBS results were examined using a range of primary thresholds (*t* = 2.5 to 3.5) to avoid false positive and bias from using a single threshold^67^. The final brain connectivity changes were presented using the results calculated at *t* = 2.8 based on the highest statistical significance (the lowest family wise error rate (FWER)-corrected *p*-value of any components). No differences in the volumetric and tractography analyses were found between sexes within each genotype in our dataset, hence results shown include combined genders.

### Immunohistochemistry

Adult mice were transcardially perfused (0.1 M phosphate buffered saline, followed by 4% paraformaldehyde (PFA)) and postfixed in 4% PFA at 4 °C as described above. Brains were removed from the skull, embedded coronally in 3% agar and sectioned at 50 µm (Leica VT1000S Vibratome). Sections at bregma level − 1.955 mm were mounted on Superfrost + slides and dried in a 37 °C oven before heat-mediated antigen retrieval (NxGen Decloaker) was performed in 10 mM sodium citrate solution (90 °C for 10 min). Fluorescence immunohistochemistry (IF) was then performed as previously described^68^. Briefly, sections were incubated overnight with primary antibodies against target proteins (Supp. Table 2). The following day, sections were rinsed in phosphate buffered saline (PBS) and then incubated with the relevant secondary antibodies for 3 h at room temperature. A list of all antibodies used is listed in Supp. Table 2. Sections were rinsed in 0.9% saline and counterstained with 4’, 6-diamidino-2-phenylindole (DAPI) and mounted in fluorescence mounting media (DAKO).

### Histological staining

Adult mice were transcardially perfused as described above. Sections at bregma levels 0.745 mm, 0.145 mm, − 0.755 mm, and − 1.955 m were stained with Haematoxylin and Eosin (H&E) using standard protocols^69^. For all H&E-stained images, sections were imaged using a brightfield slide scanner (Aperio XT Brightfield) at 40 × magnification (NA 0.75). Images were captured using ScanScope XT, and post-processed using Aperio ImageScope × 64. Images were exported as tiffs and quantified in blinded conditions (quantifications performed described in Supp. Figure 9A–D).

### Imaging and cell counts

To capture higher resolution representative images, images were acquired on a Zeiss LSM900 Airyscan 2 confocal microscope at 20x (0.8 NA) magnification. Images were acquired as WF confocal images acquired as 25 μm tiled z-stacks (0.32 μm z-step), which were stitched and rendered as maximum intensity projections using ZEN Blue software. To quantify cell number and morphology within the whole cortex, 8 μm tiled z-stacks (0.49 μm z-step) of control and cKO brains were captured at 20 × magnification (0.8 NA) using a Zeiss Axioscan Z1 slide scanner with a Colibri 7-LED light source, pentaband emission filter set (112 MBP) and Hamamatsu Orca Flash 4 sCMOS camera. The tiled z-stacks were stitched and rendered as maximum intensity projections using ZEN Blue software. All fluorophores used are provided in Supp. Table 2.

For all IF analyses except DCX, PROX1, CC3 and proliferative marker quantifications, the StarDist plugin for ImageJ^70^ was used to automate the cell count process and minimise bias. Default settings (available from StarDist’s documentation) were used for all cell counts, except for the number of tiles—this was set to 100 to accommodate whole section counts for the largest sections to be performed. Counts were performed using StarDist as previously described^71^. For DCX counts, the DG was traced and isolated from all images (encompassing dendritic area for completeness). These files were then blinded and manually quantified for soma number and occupied length. For PROX1 cell counts, the DG was traced and isolated using DAPI to guide trace thickness. These files were then blinded and manually quantified for soma number and occupied area. Cells were counted in 1000 μm^2^ grids, with 2 adjacent grids (atop each other) being quantified at 3 different positions for each blade of the DG. For CC3 cell counts, images were blinded, and both hippocampi were manually counted and quantified in Zeiss Zen Blue. For KI-67, files were blinded and manually quantified using the Cell Counter functionality within ImageJ. For co-localised proliferative cell counts, multiple channels were stacked in ImageJ (Stack functionality) and then quantified using the Cell Counter functionality. For DG- or cortex-localised SOX2^+^ cells, the DG (using DAPI as a guide) or the cortex were traced and isolated. All cells contained within the blades of the DG (as well as the hilus), or the cortex (M1) were then quantified using StarDist as described above. For hippocampal and cortically located Olig2 cells, either the hippocampus (using CA and DG as landmarks for tracing) or the cortex (M1) were traced and isolated and were then quantified using StarDist as described above.

For all histological analyses, .tiffs were blinded and quantified manually in Fiji. A description of all software used is provided in Supp. Table 3, and descriptions of all analyses completed is provided in Supp. Table 4. All analyses were completed on an n = 5 for both genotypes unless stated otherwise.

### Statistical analyses

All DTI data was validated using multiple unpaired *t*-tests—for all analyses which were analysing the same metric across different regions/structures, multiple comparisons were performed using a 5% false discovery rate (FDR), while analyses comparing different metrics across the same region/structure did not have multiple comparisons. Analyses of sex effects were performed using a 2-way ANOVA, with mice split by sex and genotype (data not shown). As no significant differences between sexes were found, sexes were pooled for all analyses. All IF and histological data were analysed using either multiple unpaired *t*-tests with Holm-Sidak correction, or Welch’s *t*-tests—multiple unpaired *t*-tests were used for all instances of multiple regions being analysed for the same measure within a graph (i.e. Fig. 5F), and Welch’s *t*-tests were used for all instances of a single measure on a single region (i.e. Fig. 5G). All data are presented as mean ± SEM. For all DTI analyses, 10 Ctrl and 9 cKO brains were used unless specified. For all histological and IF analyses, an n = 5 for both genotypes were used unless specified.

## Supplementary Information

Below is the link to the electronic supplementary material.

Supplementary Material 1

Supplementary Material 2

## Data Availability

Please contact the senior author (Michael Piper) for all data requests.
