# Supplementary material for: The histone methyltransferase SETD2 regulates adult brain structure, connectivity and neurogenesis
Source: Sci Rep. 2025 Sep 29;15:33411. doi: 10.1038/s41598-025-18780-7 (PMC12480832; doi:10.1038/s41598-025-18780-7)
Supplement: Supplementary file 2 — Supplementary Material 2 [file 41598_2025_18780_MOESM2_ESM.pdf]

SUPP FIG 1

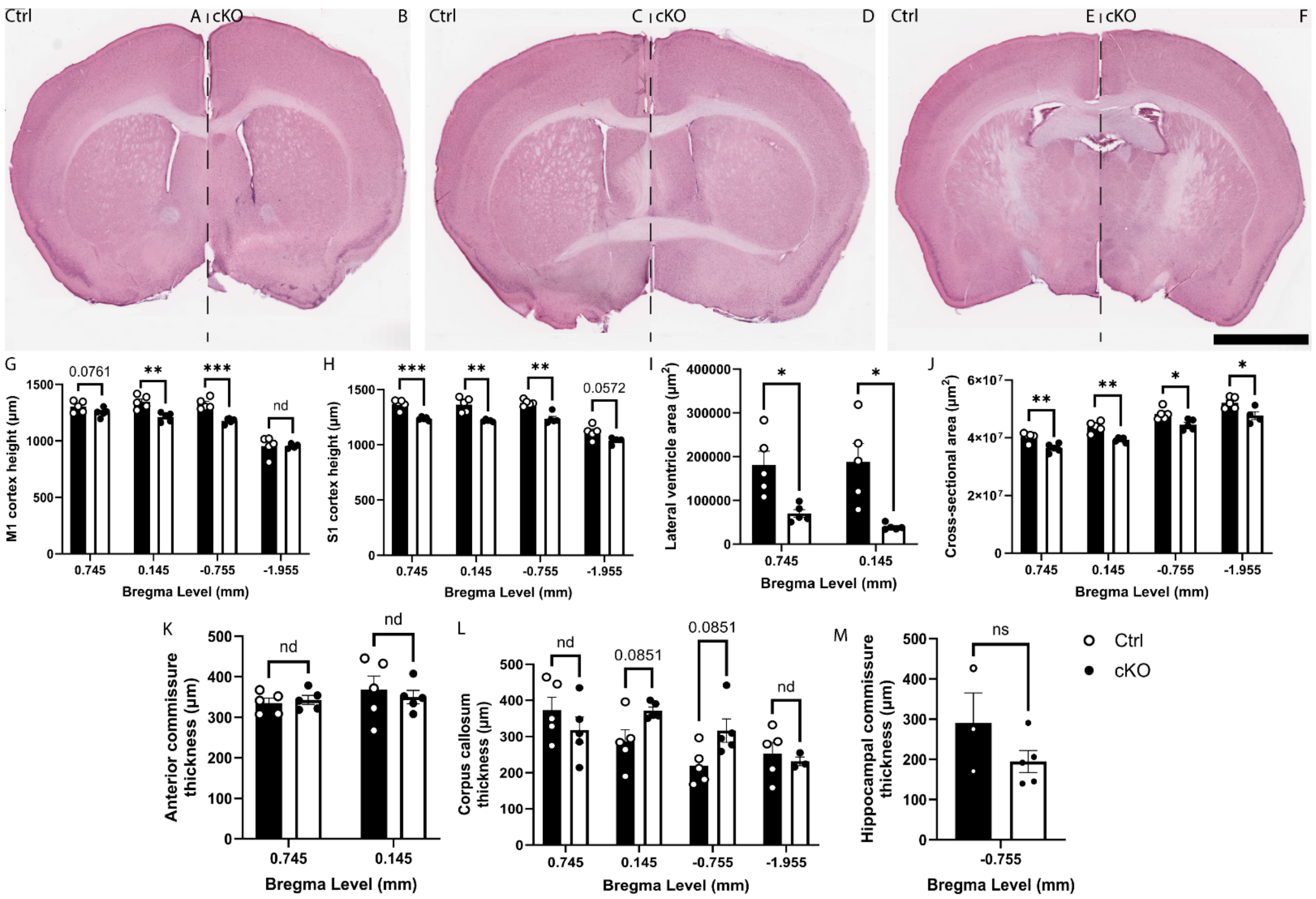

Ctrl
A
cKO
B
Ctrl
C
cKO
D
Ctrl
E
cKO
F
G
M1 cortex height (μm)
0.0761
**
***
nd
0.745
0.145
-0.755
-1.955
Bregma Level (mm)
H
S1 cortex height (μm)
***
**
**
0.0572
0.745
0.145
-0.755
-1.955
Bregma Level (mm)
I
Lateral ventricle area (μm²)
*
*
0.745
0.145
Bregma Level (mm)
J
Cross-sectional area (μm²)
**
**
*
*
0.745
0.145
-0.755
-1.955
Bregma Level (mm)
K
Anterior commissure thickness (μm)
nd
nd
0.745
0.145
Bregma Level (mm)
L
Corpus callosum thickness (μm)
nd
0.0851
0.0851
nd
0.745
0.145
-0.755
-1.955
Bregma Level (mm)
M
Hippocampal commissure thickness (μm)
ns
-0.755
Bregma Level (mm)
Ctrl
cKO

SUPP FIG 2

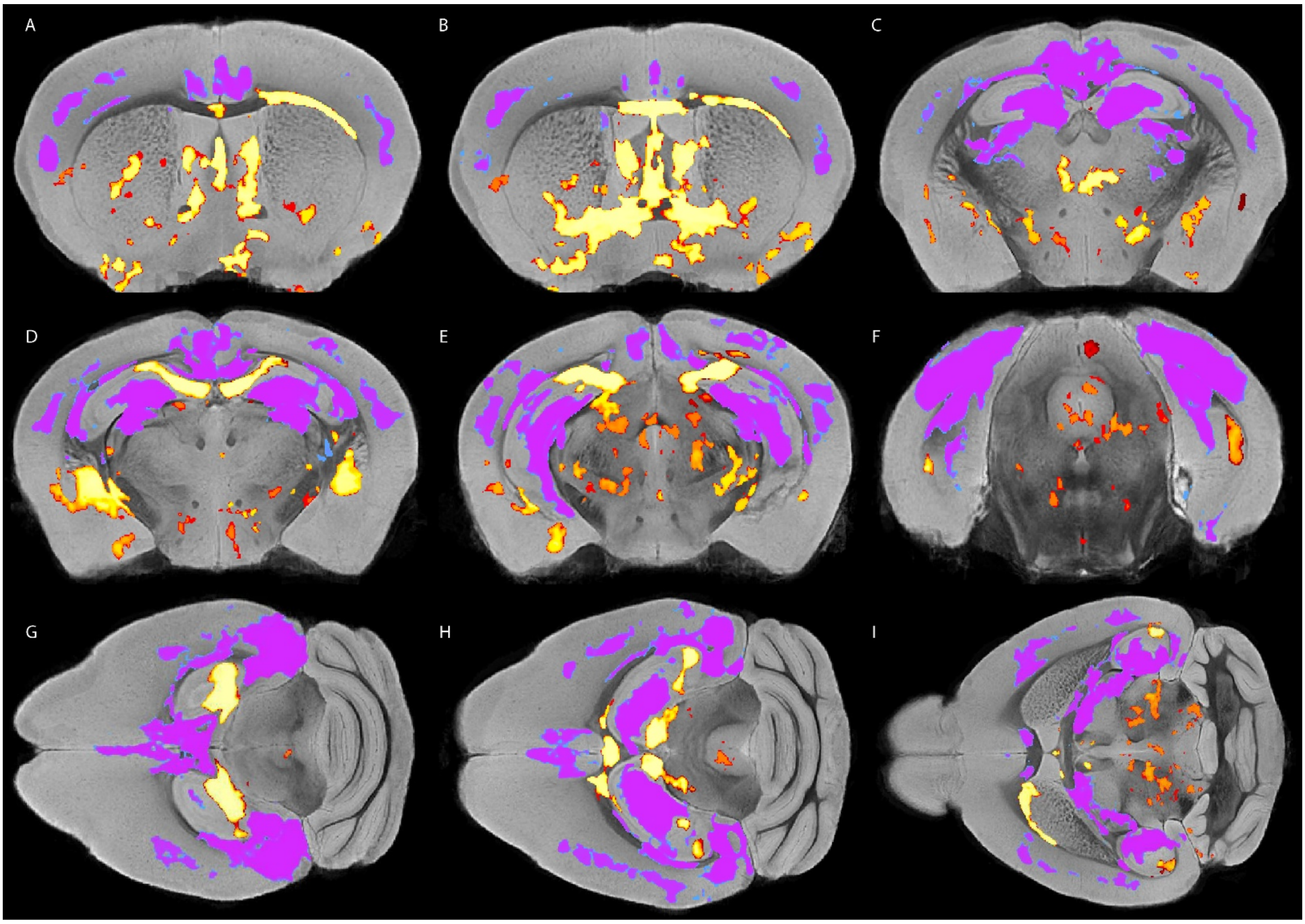

SUPP FIG 3

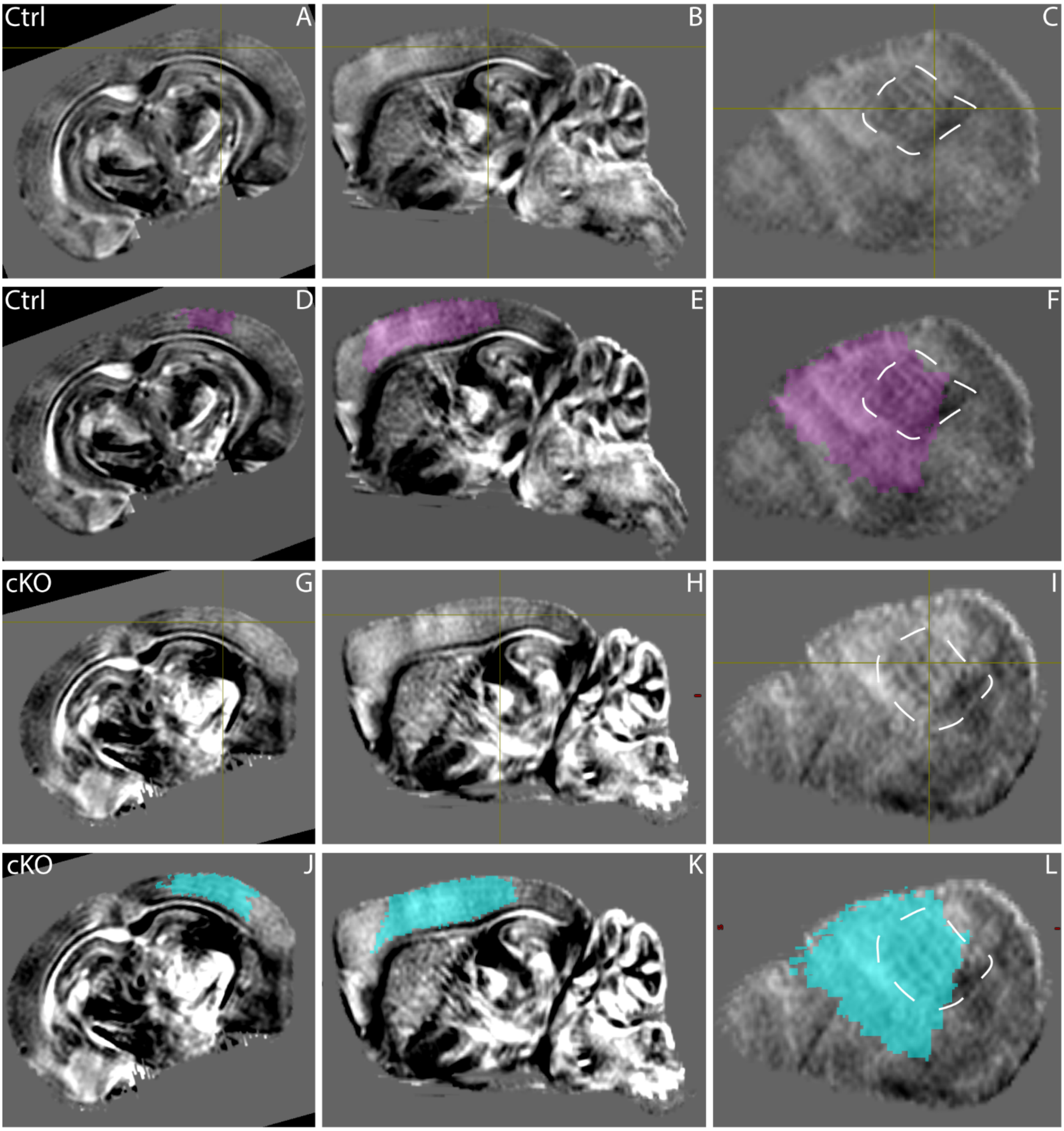

SUPP FIG 4

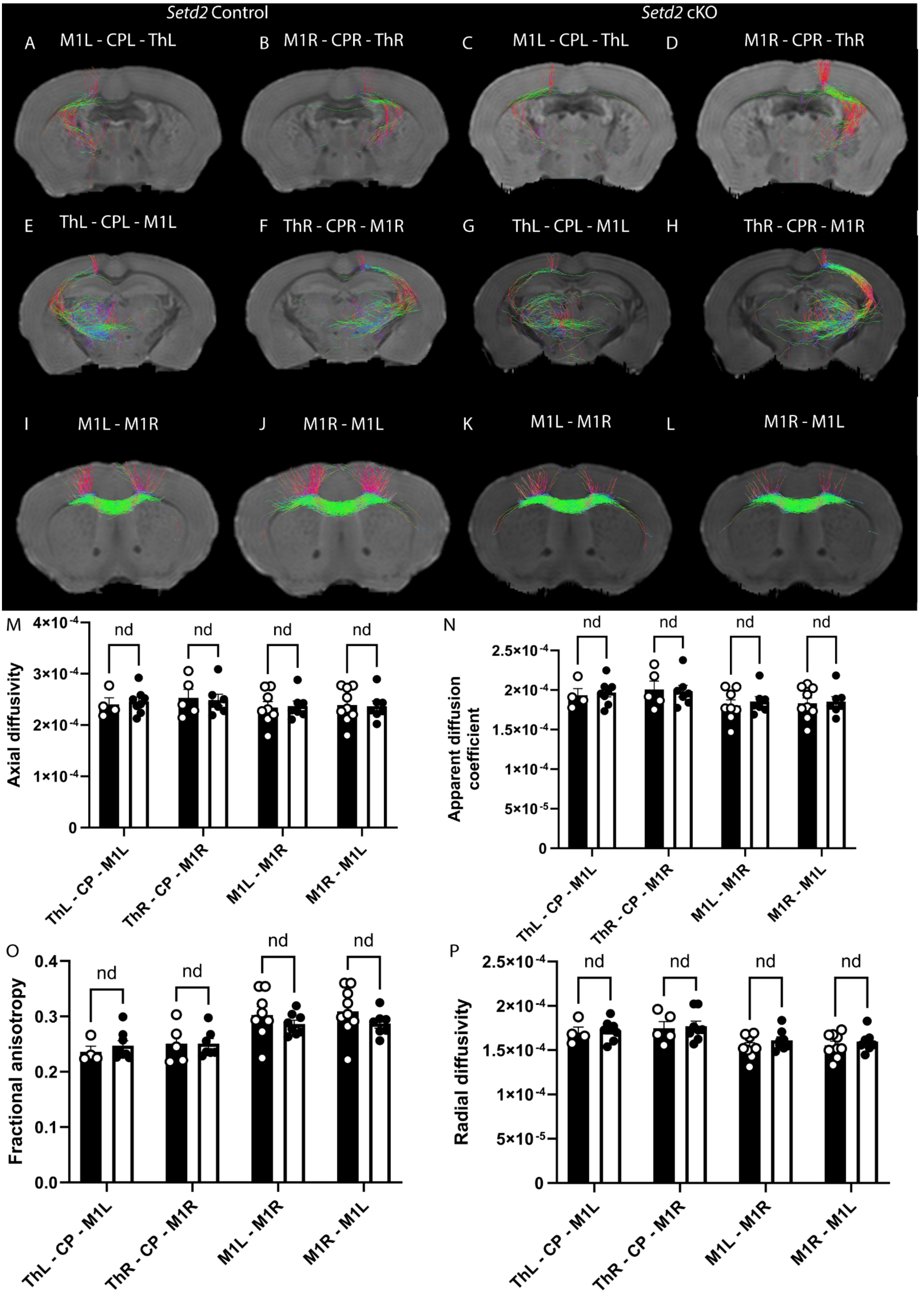

Setd2 Control
Setd2 cKO
A M1L - CPL - ThL
B M1R - CPR - ThR
C M1L - CPL - ThL
D M1R - CPR - ThR
E ThL - CPL - M1L
F ThR - CPR - M1R
G ThL - CPL - M1L
H ThR - CPR - M1R
I M1L - M1R
J M1R - M1L
K M1L - M1R
L M1R - M1L
M
Axial diffusivity
nd
ThL - CP - M1L
ThR - CP - M1R
M1L - M1R
M1R - M1L
N
Apparent diffusion coefficient
O
Fractional anisotropy
P
Radial diffusivity

SUPP FIG 5

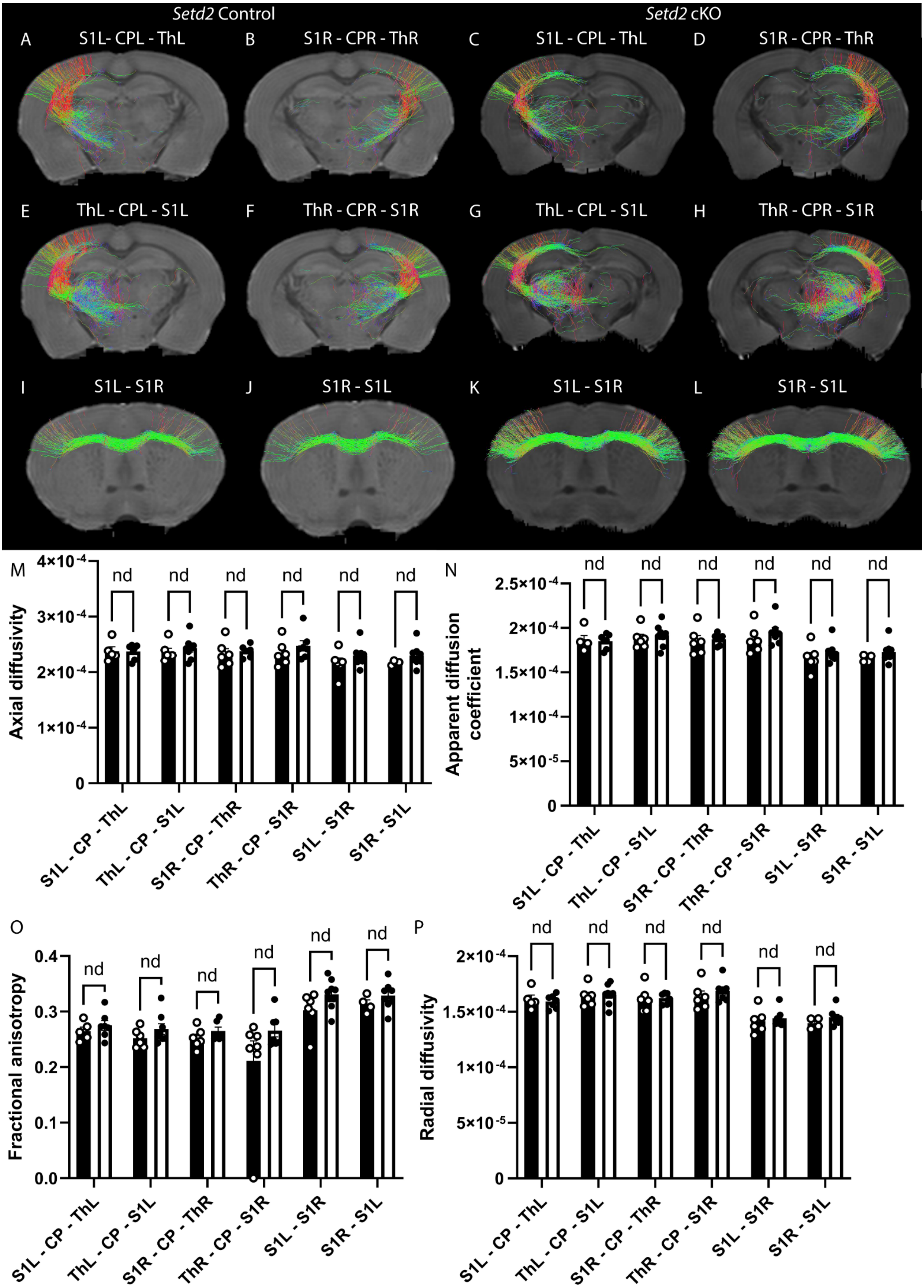
Setd2 Control
Setd2 cKO
A S1L- CPL - ThL
B S1R - CPR - ThR
C S1L - CPL - ThL
D S1R - CPR - ThR
E ThL - CPL - S1L
F ThR - CPR - S1R
G ThL - CPL - S1L
H ThR - CPR - S1R
I S1L - S1R
J S1R - S1L
K S1L - S1R
L S1R - S1L
M
Axial diffusivity
N
Apparent diffusion coefficient
O
Fractional anisotropy
P
Radial diffusivity
nd
S1L - CP - ThL
ThL - CP - S1L
S1R - CP - ThR
ThR - CP - S1R
S1L - S1R
S1R - S1L

SUPP FIG 6

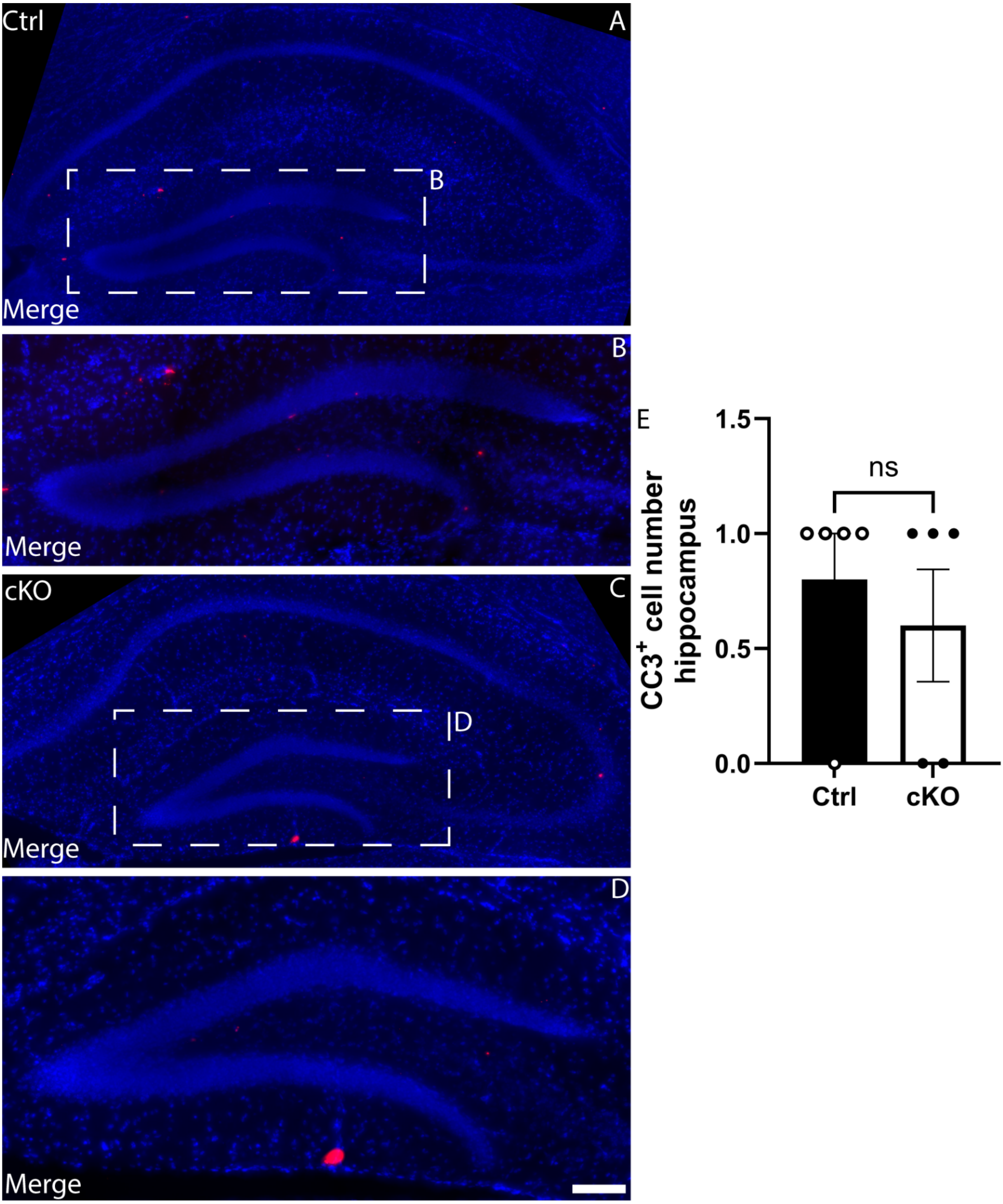

Ctrl
A
B
Merge
B
Merge
cKO
C
D
Merge
D
Merge
E
1.5
1.0
0.5
0.0
ns
CC3+ cell number hippocampus
Ctrl
cKO

SUPP FIG 7

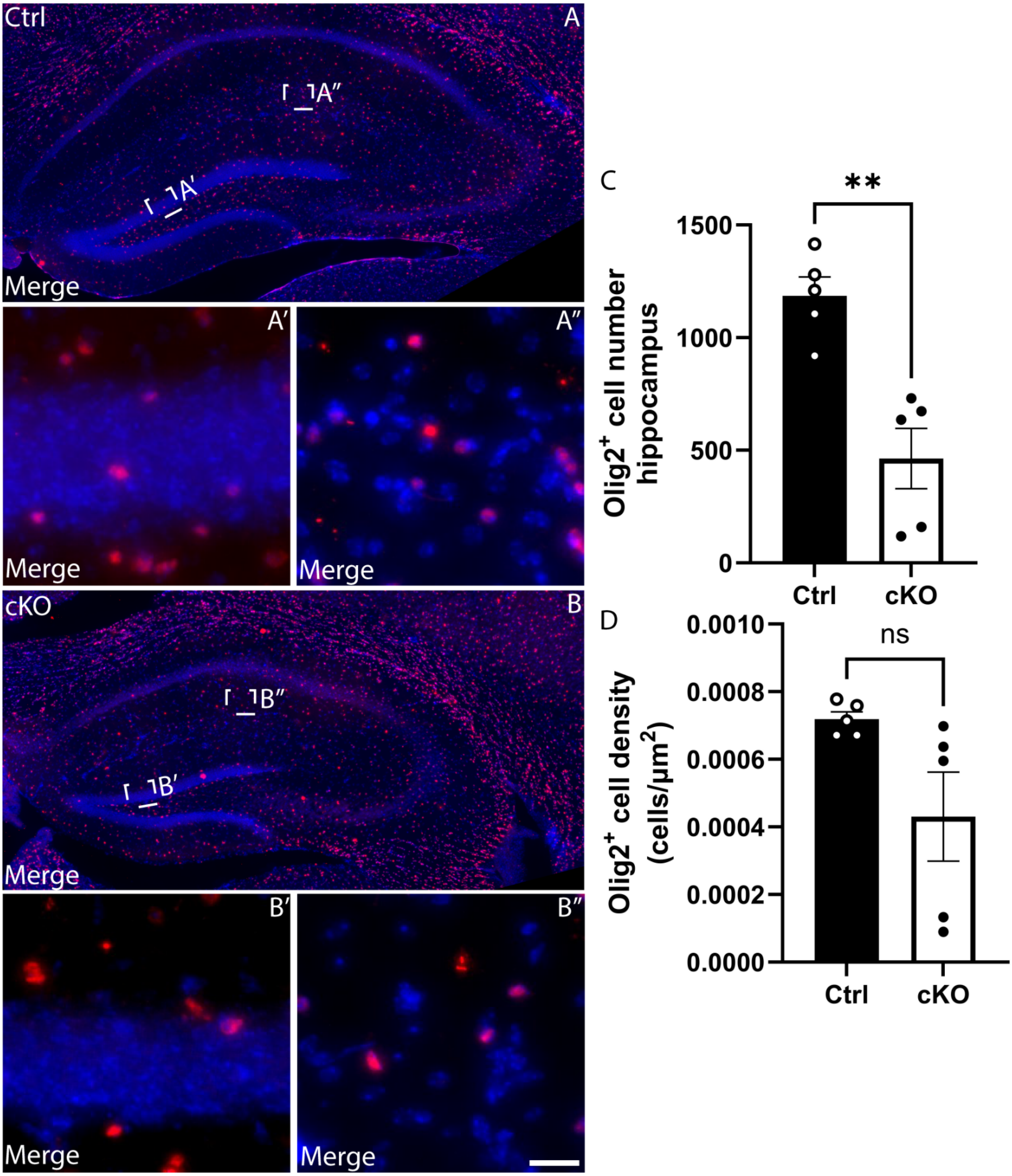
Ctrl
A
A″
A′
Merge
A′
Merge
A″
Merge
cKO
B
B″
B′
Merge
B′
Merge
B″
Merge
C
**
Olig2+ cell number hippocampus
1500
1000
500
0
Ctrl
cKO
D
ns
Olig2+ cell density (cells/µm2)
0.0010
0.0008
0.0006
0.0004
0.0002
0.0000
Ctrl
cKO

SUPP FIG 8

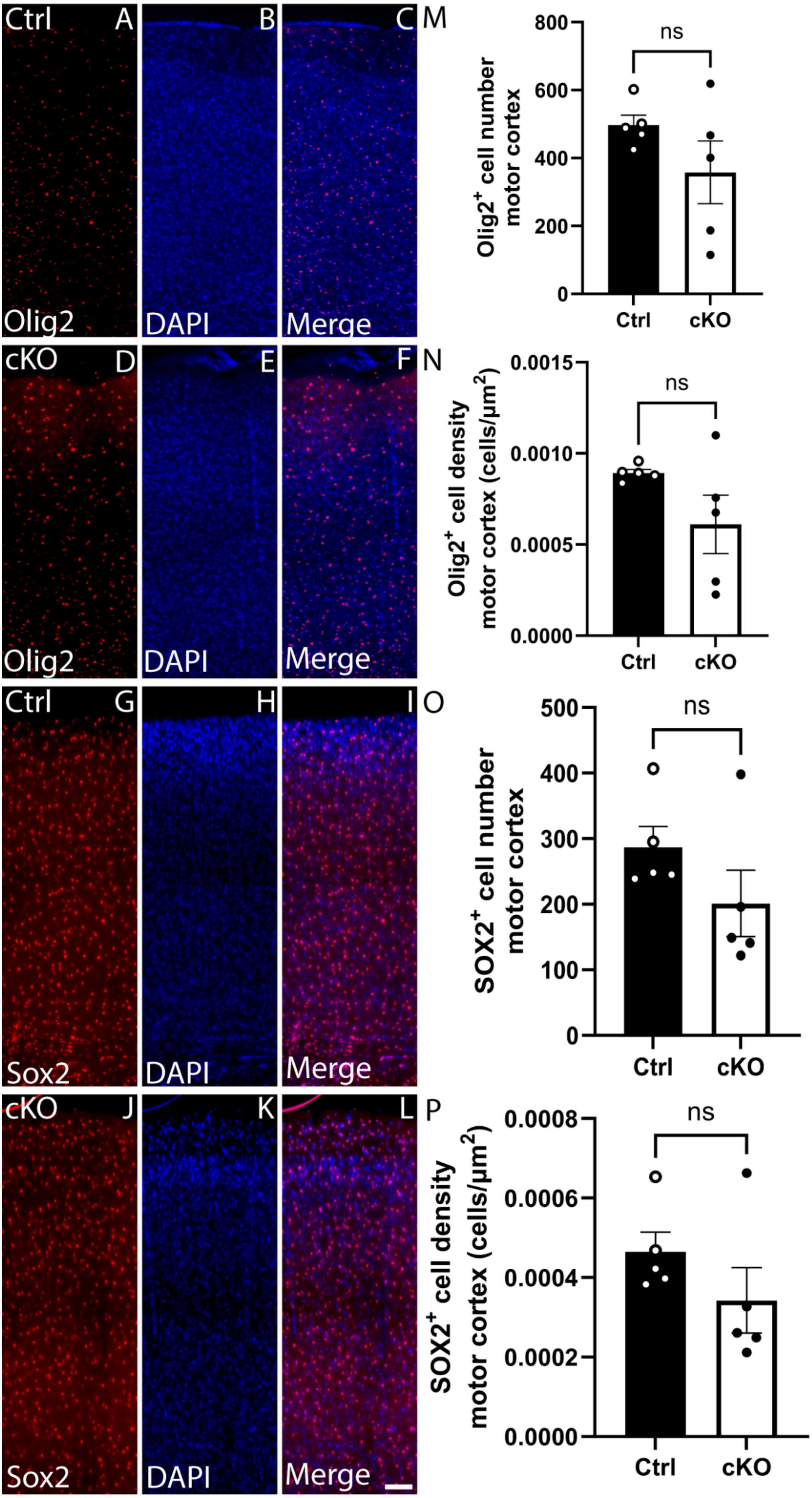
Ctrl
A
B
C
M
Olig2
DAPI
Merge
cKO
D
E
F
N
G
H
I
O
Sox2
J
K
L
P
Olig2+ cell number motor cortex
Olig2+ cell density motor cortex (cells/µm2)
SOX2+ cell number motor cortex
SOX2+ cell density motor cortex (cells/µm2)
ns
800
600
400
200
0
0.0015
0.0010
0.0005
0.0000
500
300
100
0.0008
0.0006
0.0004
0.0002

SUPP FIG 9

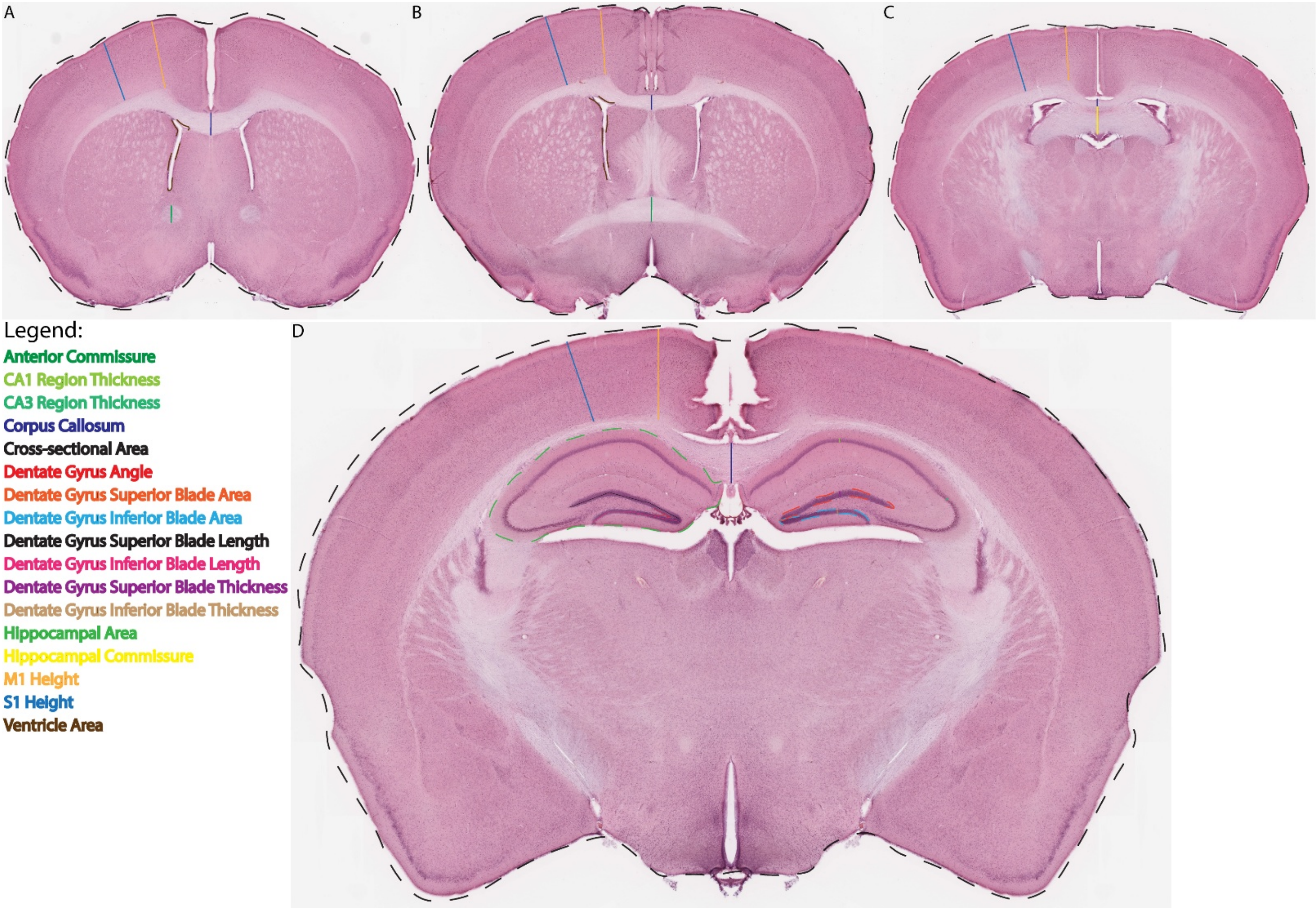
A
B
C
D
Legend:
Anterior Commissure
CA1 Region Thickness
CA3 Region Thickness
Corpus Callosum
Cross-sectional Area
Dentate Gyrus Angle
Dentate Gyrus Superior Blade Area
Dentate Gyrus Inferior Blade Area
Dentate Gyrus Superior Blade Length
Dentate Gyrus Inferior Blade Length
Dentate Gyrus Superior Blade Thickness
Dentate Gyrus Inferior Blade Thickness
Hippocampal Area
Hippocampal Commissure
M1 Height
S1 Height
Ventricle Area

SUPPLEMENTARY FIGURE LEGENDS

Supp Fig 1: H&E morphological analyses reveal structurally reduced area and widths largely in agreement with volumetric analyses. (A-F) Haematoxylin and eosin (H&E) stained sections taken across the rostrocaudal axis (bregma's include 0.745mm, 0.145mm, -0.755mm and -1.955mm) of control (A, C, E) and cKO (B, D, F) brains. Quantification of G) primary motor cortex height, H) primary somatosensory cortex height, I) lateral ventricle area, J) cross-sectional area (of each entire section), K) anterior commissure thickness, L) corpus callosum thickness and M) hippocampal commissure thickness revealed significant reductions throughout the rostrocaudal axis of the brain in several different structures. Scale bar in F represents 2mm for all images. For all analyses, an $n = 5$ was used for each genotype, except for bregma level -1.955mm, which had $n = 4$ for cKO brains. nd = no difference, ns = not significant, * = $p < 0.05$, ** = $p < 0.01$, *** = $p < 0.001$.

Supp Figure 2: Tensor-based morphometry reveals global alterations to structural volumes across *Setd2* cKO brains. Tensor based morphometry (TBM) analyses are shown in representative average-brain templates for coronal (A-F) and transverse (G-I) planes, highlighting differences in structural volume in cKO brains overlaid over Ctrl brains. TBM analyses revealed decreased (Ctrl > cKO) average structural volume (purple scale) predominantly within grey matter substructures, while increased (Ctrl < cKO) average structural volumes (orange scale) were found within both white and grey matter substructures. These structural volume alterations are represented across the rostrocaudal axis (with A being most rostral, and F being most caudal), as well as along the dorsoventral axis (with G being most dorsal and I being most ventral).

Supp Fig 3: Mapping efficacy is similar despite arealisation defects. Constrained spherical deconvolution (CSD) map images (with coefficients in the order of lmY(2,1)) representing Ctrl (A-F) and cKO (G-L) brains, highlighting the localisation of the barrel cortex. CSD maps are shown in coronal (A, D, G, J), sagittal (B, E, H, K) and transverse (C, F, I, L) planes to highlight barrel cortex location. (A-C, G-I) Ctrl and cKO barrel cortices appear similarly localised, with some slight caudomedial shifts noted. (D-F, J-L) When an ROI generated to encompass S1 was overlaid on the barrel cortex, a similar proportion was covered by this ROI, suggesting that software used to generate these ROIs, and all subsequent analyses stemming from them, is reasonably accurate at mapping to the altered regions of the cKO neocortex despite arealisation defects.

Supp Fig 4: Primary motor tracks are unaffected in cKO brains. Tractography between the primary motor cortices, the caudate putamen and the thalamus (A-D), the thalamus, caudate putamen and the primary motor cortices (E-H) and between the motor cortices themselves (I-L) reveal no grossly altered tract directionality between control (A,B, E, F, I, J) and cKO (C, D, G, H, K, L) brains. Analysis of axial diffusivity (M), apparent diffusion coefficient (N), fractional anisotropy (O) and radial diffusivity (P) revealed no alterations between the selected tracks either. Analyses of M1 – Cp – Th (for both left and right tracks) could not be performed, due to insufficient sample sizes. Multiple unpaired t-tests with a 5% FDR were used for all statistics, minimum of n = 4 used for all samples. Colours for DTI represent the following directionality: green is left-right oriented, red is dorso-ventral oriented, blue is rostro-caudal oriented; oblique fibre orientations are a combination of these colours. nd = no difference.

Supp Fig 5: Primary somatosensory tracks are unaffected in cKO brains. Tractography between the primary somatosensory cortices, the caudate putamen and the thalamus (A-D), the thalamus, caudate putamen and the primary somatosensory cortices (E-H) and between the somatosensory cortices themselves (I-L) reveal no grossly altered tract directionality between control (A,B, E, F, I, J) and cKO (C, D, G, H, K, L) brains. Analysis of axial diffusivity (M), apparent diffusion coefficient (N), fractional anisotropy (O) and radial diffusivity (P) revealed no alterations between the selected tracks either. Multiple unpaired t-tests with a 5% FDR were used for all statistics, minimum of n = 4 used for all samples. Colours for DTI represent the following directionality: green is left-right oriented, red is dorso-ventral oriented, blue is rostro-caudal oriented; oblique fibre orientations are a combination of these colours. nd = no difference.

Supp Fig 6: Apoptosis is not elevated in the hippocampus of adult *Setd2* cKO mice. Coronal sections taken at the level of the dentate gyrus of the hippocampus from adult control (A-C) and cKO (D-F) brains, revealing the expression of CC3 (red) and DAPI (blue). Quantification of G) the number of CC3+ cells (DAPI-localised) within the hippocampus reveals no elevation of CC3+ apoptotic cells. ns = not significant. Scale bar in F represents 200 µm for all panels. For analyses in E, an n = 5 was used for both genotypes.

Supp Figure 7: *Setd2* cKO brains show alterations in hippocampal oligodendrocyte populations. Coronal sections taken at the level of the dentate gyrus of the hippocampus from adult control (A-C) and cKO (D-F) brains, revealing the expression of Olig2 (red) and DAPI (blue). Quantification of G) the number of Olig2+ cells within the DG, and H) density of those cells in the DG. ns = not significant; ** $p < 0.01$. Scale bar in F represents 200 µm for A, B and 10 µm for all zooms. For all analyses, an n = 5 was used for both genotypes.

Supp Fig 8: Non-neuronal cell populations are unaffected in the cortex of *Setd2* cKO mice. Coronal sections taken at the level of the hippocampus from adult primary motor cortices, revealing the expression of Olig2 (red, A-F), SOX2 (red, G-L) and DAPI (blue) within control (A-C, G-I) and cKO (D-F, J-L) brains. Quantification of Olig2+ or SOX2+ cell number (M and O respectively), or the density of these cells (N and P respectively) reveals no differences within these populations. ns = not significant. Scale bar in F represents 100 µm for all panels. For all analyses, an n = 5 was used for each genotype.

Supp Fig 9: Schematic overview of how H&E morphological analyses were performed. A-D) Histological sections showing H&E-stained control brains, representative of sections used for morphological analyses. Coloured lines are overlaid on each section, highlighting how a specific measure was performed – each colour then corresponds to one measure, specified in the legend (left). Where a certain measure was repeated across multiple bregma's (i.e. cross-sectional area), the same colour is used across sections. All sections used in this figure were taken from Fig. 5 and Supp. Fig. 1. For all analyses, an n = 5 was used for each genotype.

SUPPLEMENTARY TABLES

Supp Table 1: Details of all primer sequences used for genotyping.

PRIMER SEQUENCES	
**Primer Type**	**Primer Sequence**
SETD2 Forward	5' – TTACTAGCTGTGAGTCACTGATTCC
SETD2 Reverse	5' – TCCAAGATGCATATTCTCTACCTG
Emx1 Forward	5' – GAGGGACTACCTCCTGTACC
Emx1 Reverse	5' – TGCCCAGAGTCATCCTTGGC

Supp Table 2: Details of all antibodies used in this study for IF analyses.

PRIMARY ANTIBODIES					
Antibody	Species	Company	Catalog #	Dilution	Fluorophore
GFAP	Mouse	MilliporeSigma	MAB360	1/500	AF488
SOX2	Rat	eBioscience	14-9811-82	1/500	AF488, AF555
DCX	Goat	Santa Cruz	sc-8066	1/400	AF647
Ki67	Mouse	BD Biosciences	550609	1/400	AF647
CC3	Rabbit	Cell Signalling Technology	9661S	1/400	AF750
Olig2	Rabbit	Sigma Aldrich	AB9610	1/400	AF750
PROX1	Rabbit	Abcam	ab199359	1/400	AF750
SECONDARY ANTIBODIES & DAPI					
Mouse α 488	Donkey	Jackson ImmunoResearch	715-546-150	1/1000	
Rat α 488	Donkey	Jackson ImmunoResearch	712-546-150	1/1000	
Rabbit α 555	Donkey	Jackson ImmunoResearch	711-165-152	1/1000	
Rat α 555	Goat	Thermofisher	A-21434	1/1000	
Goat α 647	Donkey	Jackson ImmunoResearch	705-607-003	1/1000	
Mouse α 647	Donkey	Jackson ImmunoResearch	715-605-150	1/1000	
Goat α 750	Donkey	Abcam	ab175744	1/1000	
Rabbit α 750	Donkey	Abcam	ab175731	1/1000	
DAPI	N/A	Thermofisher	D1306	1/500	

Supp Table 3: Details of all software used for analyses.

Software Package Name	Software Package Source	Software Package Purpose
FSL	https://fsl.fmrib.ox.ac.uk/fsl/fslwiki	Linear atlas registration to cerebellum
ANTS	https://stnava.github.io/ANTs/	Warped/deformation atlas registration to cerebellum
ITKSnap	http://www.itksnap.org/pmwiki/pmwiki.php	Segment structures and adjust templates ROIs to match anatomical regions
MRtrix3	www.mrtrix.org	Diffusion MRI data analyses
GraphPad Prism	N/A	Statistical analyses
MATLAB 2020b	N/A	NBS Analyses
Zeiss Zen Blue	N/A	Processing and image analysis of all immunohistochemical images
Fiji (ImageJ)	N/A	Post-processing and analyses of all histological and immunohistochemical data
Aperio ImageScope x64	N/A	Analyses of all brightfield histological sections

Supp Table 4: Details of all analyses performed.

Analysis Performed	Analysis Details	Software Used for Analysis
Histological Analyses (H&E-stained sections)	All analyses were performed blinded using Fiji for quantification. Standard in-built features of Fiji only used for analyses	Fiji, Aperio ImageScope x64
SOX2 IF Cell Counts (DG)	Traced the dentate gyrus using PROX1 as an overlay to determine DG area (including all cells in between the blades (hilus))<br>Isolated SOX2 from PROX1, and quantified SOX2 number using StarDist (standard with tiles set = 100)	Fiji, Zen Blue
SOX2 IF Cell Counts (Cortex)	Isolated 500 µm width column of primary motor cortex<br>Quantified SOX2 number using StarDist (standard with tiles set = 100)	Fiji, Zen Blue
DCX IF Cell Counts (DG)	Isolated DCX channel and performed z-projection to capture all information<br>Manually measured DCX cell soma number and occupied blade length blinded	Fiji, Zen Blue
PROX1 IF Cell Counts (DG)	Isolated PROX1 channel and single z-stack (from middle of stack for in-focus information)<br>Manually quantified (blinded) 2x 1000 μm^2 grids per region, 3 regions per blade for both blades	Fiji, Zen Blue
Olig2 IF Cell Counts (Hippocampus)	Exported both DAPI and Olig2 channels, individually performed z-projections to capture all information and merged the channels<br>Traced hippocampus (CA regions to DG, then to the midline) and isolated HC regions<br>Isolated Olig2 and quantified cell number using StarDist (standard with tiles set = 100)	Fiji, Zen Blue

Olig2 IF Cell Counts (Cortex)	Isolated 500 μm width column of primary motor cortex<br>Quantified Olig2 number using StarDist (standard with tiles set = 100)	Fiji, Zen Blue
CC3 IF Cell Counts (Hippocampus)	Manually quantified blinded images in Zen Blue for $CC3^+$ cells (localized to a $DAPI^+$ cell) within the hippocampus (CA regions to DG, then to the midline inclusive)	Zen Blue
Ki67 IF Cell Counts (DG)	Manually quantified blinded images, counting each channel individually. All channels were quantified as maximum intensity projections of the entire z-stack and counted using Cell Counter. Only cells which sat within the SGZ (demarcated by DAPI) were quantified.	Fiji, Zen Blue
Sox2/Ki67 Double Pos IF Cell Counts (DG)	Manually quantified blinded images, counting each channel individually. All channels were quantified as maximum intensity projections of the entire z-stack and counted using Cell Counter. Only cells which sat within the SGZ (demarcated by DAPI) were quantified. Stack functionality was used to overlay counted channels and confirm double pos or single pos cells	Fiji, Zen Blue
GFAP IF Radial Fibre Counts (DG)	Manually quantified blinded images, counting each channel individually. All channels were quantified as maximum intensity projections of the entire z-stack and counted using Cell Counter. Only cells which sat within the DG (demarcated by DAPI) were quantified if they exhibited a radial fibre morphology (fibre crossing from SGZ towards the edge of the blade).	Fiji, Zen Blue
